# Cotton pedigree genome reveals restriction of cultivar-driven strategy in cotton breeding

**DOI:** 10.1186/s13059-023-03124-3

**Published:** 2023-12-08

**Authors:** Shang Liu, Dongyun Zuo, Hailiang Cheng, Man He, Qiaolian Wang, Limin Lv, Youping Zhang, Javaria Ashraf, Ji Liu, Guoli Song

**Affiliations:** 1 Institute of Cotton Research of Chinese Academy of Agricultural Sciences, Anyang, 455000 China; 2 Zhengzhou Research Base, State Key Laboratory of Cotton Biology, Zhengzhou University, Zhengzhou, 450001 China; 3 Department of Plant Breeding and Genetics, Faculty of Agriculture and Environment, The Islamia University of Bahawalpur, Bahawalpur, Pakistan

**Keywords:** Cotton pedigree, Nanopore long read, Graphical genome, Cultivar-driven strategy

## Abstract

**Background:**

Many elite genes have been identified from the available cotton genomic data, providing various genetic resources for gene-driven breeding. However, backbone cultivar-driven breeding is the most widely applied strategy. Revealing the genetic basis of cultivar-driven strategy’s restriction is crucial for transition of cotton breeding strategy.

**Result:**

CRI12 is a backbone cultivar in cultivar-driven breeding. Here we sequence the pedigree of CRI12 using Nanopore long-read sequencing. We construct a graphical pedigree genome using the high-quality CRI12 genome and 13,138 structural variations within 20 different pedigree members. We find that low hereditary stability of elite segments in backbone cultivars is a drawback of cultivar-driven strategy. We also identify 623 functional segments in CRI12 for multiple agronomic traits in presence and absence variation-based genome-wide association study on three cohorts. We demonstrate that 25 deleterious segments are responsible for the geographical divergence of cotton in pathogen resistance. We also characterize an elite pathogen-resistant gene (GhKHCP) utilized in modern cotton breeding. In addition, we identify 386 pedigree fingerprint segments by comparing the segments of the CRI12 pedigree with those of a large cotton population.

**Conclusion:**

We characterize the genetic patterns of functional segments in the pedigree of CRI12 using graphical genome method, revealing restrictions of cultivar-driven strategies in cotton breeding. These findings provide theoretical support for transitioning from cultivar-driven to gene-driven strategy in cotton breeding.

**Supplementary Information:**

The online version contains supplementary material available at 10.1186/s13059-023-03124-3.

## Background

Upland cotton (*Gossypium hirsutum* L.) is an important economic crop due to its high yield and quality of natural fibers [1, 2]. Therefore, one of the primary goals for cotton breeders is to create new lines that can effectively enhance fiber yield and quality. In cotton breeding practices, an accession with elite integrative traits is used as the backbone and crossed with other cultivars to create new lines which is a cultivar-driven strategy [3, 4]. For instance, CRI12, as a cultivar, won the first prize of the National Award for Technological Invention of China, is selected from cross lines in disease nursery, and has elite integrative agronomic traits, especially for high resistance to *Fusarium wilt* and *Verticillium wilt*. This backbone cultivar is used to create many progenies, forming CRI12 pedigree by the cultivar-driven strategy [4]. However, progenies in CRI12 pedigree do not retain elite integrative agronomic traits. Apart from CRI12, many other popular cultivars were also used to create new lines in cotton breeding history, and they exhibited limited effectiveness in cultivar-driven breeding strategy which appeared in CRI12 pedigree [5]. Although these popular backbone cultivars are focused by both cotton genomics community and cotton breeders, genetic basis of cultivar-driven strategy’s restriction in cotton breeding history is still mysterious [3–6].

With a large number of cotton accessions resequenced, a diverse gene pool related to lint percentage (LP), fiber length (FL), fiber strength (FS), and other agronomic traits has emerged [3–6]. More published cotton reference genomes bring cotton genomics to a new era in which pan-genome comes with attention on structural variation (SV) due to its large genetic effects and strong interpretability in non-coding regions [7–13]. Graph-based cotton pan-genome detected new valuable genes from previous cohorts, proving the utility of SVs in cotton genomics [11]. However, these genomic analyses based on natural population, genetic population, and even cotton pedigrees always focused on identification of functional genes and ignored the link between genomic analysis (gene-driven) and breeding strategy (cultivar-driven), resulting in significant gap between cotton genomics and cotton breeding practices.

**Fig. 1:**
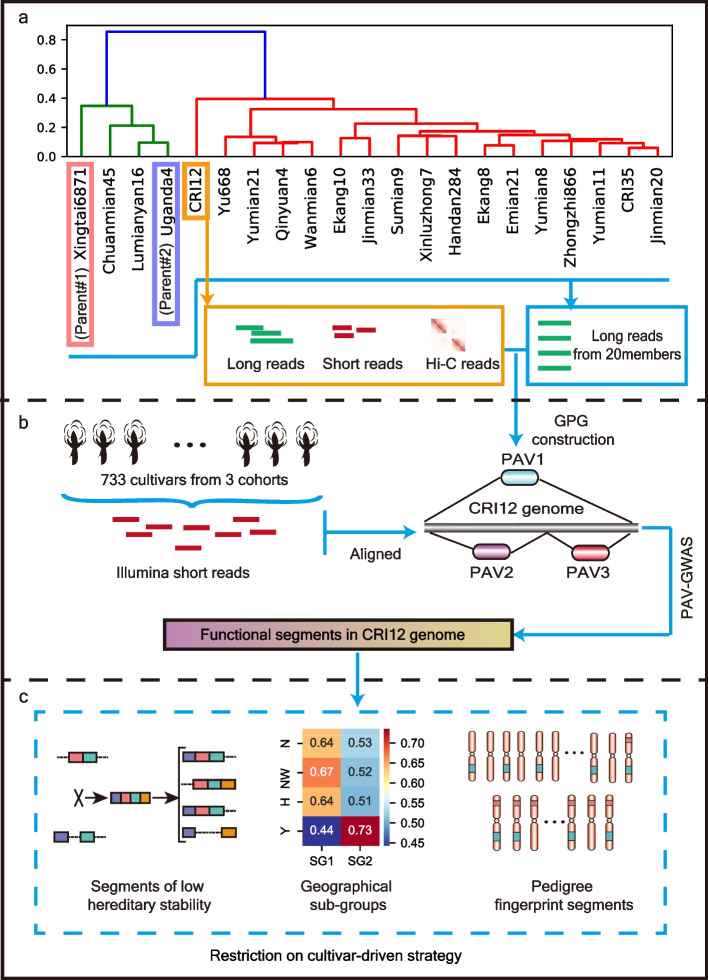
Overview of this study. **a** Illustration for sampling and sequencing for CRI12 and its pedigree members. Nanopore long reads from 20 pedigree members were gained. **b** PAVs were used to build a graph-genome which was used for PAV-GWAS analysis based on 3 cohorts. **c** Segments of low hereditary stability, geographical sub-groups in functional segments, and pedigree fingerprint segments were investigated based on functional segments in CRI12 genome

To understand genetic mechanisms underlying restrictions of cultivar-driven strategy and promote breeding approach shift to gene-driven strategy, we collected CRI12 and its 20 pedigree members (comprising 2 parents and 18 progenies). High-quality CRI12 genome was assembled and its 20 pedigree members were sequenced by long-read sequencing technology, giving approximately 20 × depth per sample (Fig. 1a). After integrating pedigree variations into a graph-based genome, we utilized three previous cohorts (Wang_2017, Ma_2018, and He_2021; *n* = 733) to identify functional variations via presence/absence variation-based genome-wide association study (PAV-GWAS) (Fig. 1b) [14–16]. Based on the results of the PAV-GWAS, we found restrictions on cultivar-driven strategy exploring three aspects, (i) segments of low hereditary stability; (ii) geographical sub-groups; (iii) pedigree fingerprint segments (Fig. 1c). The cotton pedigree genome informs us to understand genetic basis of limited effectiveness in cultivar-driven breeding strategy and provides theorical support for breeding strategy shift.

## Results

### Characterization of genomic variations in CRI12 pedigree

**Fig. 2:**
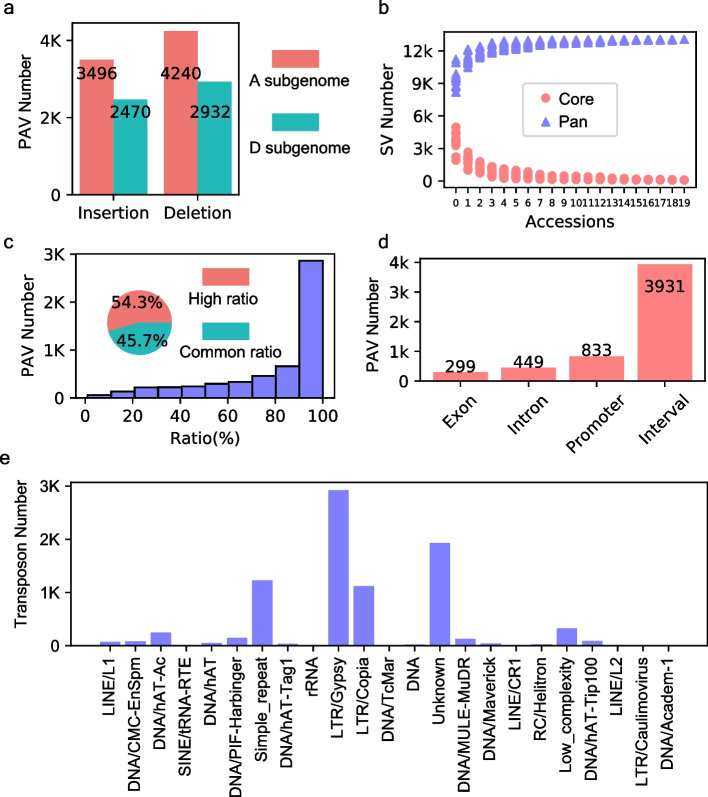
Characterization of genomic variations in CRI12 pedigree. **a** Statistic about number of PAVs identified in CRI-12 pedigree (insertions and deletions) within A and D sub-genomes. **b** Saturation curve of core and pan SVs in the CRI12 pedigree with cultivars added. **c** Statistic about PAV sequences influenced by transposons. Barplot was the distribution of transposon ratio in transposon-influenced segments. Pie plot represents PAVs of high transposon ratio (≥ 90%) and common transposon ratio (< 90%). **d** Genomic annotation of transposon-influenced segments. **e** Statistic about number of transposons of various categories in pedigree

We employed a combination of Nanopore long reads, Illumina short reads, and Hi-C data to assemble the CRI12 genome, capturing a total of 2.24 Gb sequences (contig N50 = 11.2 Mb) (Table 1; Additional file 1: Table S1-S3). The assembled genome exhibited high syntenic ratios (ranging from 93.5 to 98.5%) with other upland cotton genomes (Additional file 1: Table S4) [7, 8, 17]. Moreover, we evaluated the completeness of the CRI12 genome using Benchmarking universal single-copy orthologs (BUSCO) and Core Eukaryotic Genes Mapping Approach (CEGMA), achieving completeness of 96.1 and 95.56%, respectively [18, 19] (Table 1) [7, 8, 17]. We annotated a total of 72,262 protein-coding genes after conducting continuity, consistency, and completeness (3C) evaluations of the assembled CRI12 genome (Table 1). Our results suggest that the CRI12 genome assembly is highly competent and can be used as a reference genome for downstream analyses. To investigate genetic pattern of segments from CRI12 in cultivar-driven strategy, we collected 20 CRI12 pedigree members including CRI12’s parents ( Xingtai6871 and Uganda4; defined as Parent#1 and Parent#2 in this study, respectively) and 18 progenies for whole-genome sequencing using ONT long reads to a median of 20 × coverage (Fig. 1a; Additional file 1: Table S5-S6). We identified a total of 13,138 high-quality PAVs (7172 deletions and 5966 insertions) after aligning the long reads of the pedigree members to the CRI12 genome (Fig. 2a; Additional file 1: Table S7). Upland cotton is an allotetraploid, and its genome (AADD) consists of sub-genome A and sub-genome D. In CRI12 pedigree, there is no preference on insertion or deletions between A and D sub-genomes (Fig. 2a; *P* = 0.55, *χ*^2^ test). The pan-core curve of the 13,138 PAVs eventually reached a plateau, confirming the representativeness of the 20 pedigree members we selected (Fig. 2b).

**Table 1:** BUSCOs and CEGMAs indicated ratios of benchmarking genes in 2 pipelines were correctly assembled in CRI12 genome. The ratios could represent completeness of assembled genome. Repeat ratio indicated ratio of repeat sequence in CRI12 genome

Category	CRI12 genome
Genome size	2239 Mb
Contigs	974
ContigN50	11.2 Mb
Scaffolds	270
ScaffoldN50	91 Mb
GC content	34.16%
Gene numbers	72,262
BUSCOs	96.10%
CEGMAs	95.56%
Repeat ratio	62.75%

Previous studies have demonstrated that transposon activity drives genomic variation, thereby promoting genetic innovation [10, 20, 21]. In this study, we annotated sequences of 13,138 PAVs, 6076 among them were generated through transposition events (Additional file 1: Table S8). A considerable proportion (54.3%) of the transposon-influenced PAVs displayed a high transposon ratio (> 90%), showing transposons also contribute to genomic variations in pedigree (Fig. 2c). Furthermore, we found that exons and introns exhibited fewer transposon activities (299 in exons and 449 in introns) as compared to interval and promoter, i.e., 3931 in interval and 833 in promoter, indicating genes tend to repel transposon activity (Fig. 2d). Analysis of transposon categories revealed a total of 26 distinct categories found within the 6076 transposon-influenced PAVs. Of these categories, LTR/Gypsy, the most abundant transposon, is highly related with PAV length in exon, promoter, and interval regions (Pearson correlation is 0.81, 0.72, and 0.73, respectively; 5627 of 6076 PAVs) and relatively lowly related with PAV length in intron (Pearson correlation is 0.64; 449 of 6076 PAVs) (Fig. 2e; Additional file 2: Fig. S1a), indicating its important role in SVs proved in *G. rotundifolium* [20]. Surprisingly, we observed a strong correlation (*r*^2^ = 0.85) between PAV length in exons and the number of Simple_repeats, which were present at a notably higher proportion in exons (19%) when compared to interval regions (13%) (Additional file 2: Fig. S1b). These findings suggest that Simple_repeat is highly correlated with exon, and their relationship should be further investigated in future studies. Overall, we constructed a high-quality PAV map which could present pedigree’s variation, providing a platform for functional interpretation on CRI12 pedigree’s large sequence variations.

### CRI12 pedigree contains valuable genomic variations

SVs contribute significantly to phenotype during crop breeding. In cotton, the use of an SV-based pan-genome has accurately reflected the breeding history in China. To identify valuable SVs during the formation of the CRI12 pedigree, we constructed a Graphical Pedigree Genome (GPG) by integrating 13,138 PAVs into the CRI12 reference genome.

**Fig. 3:**
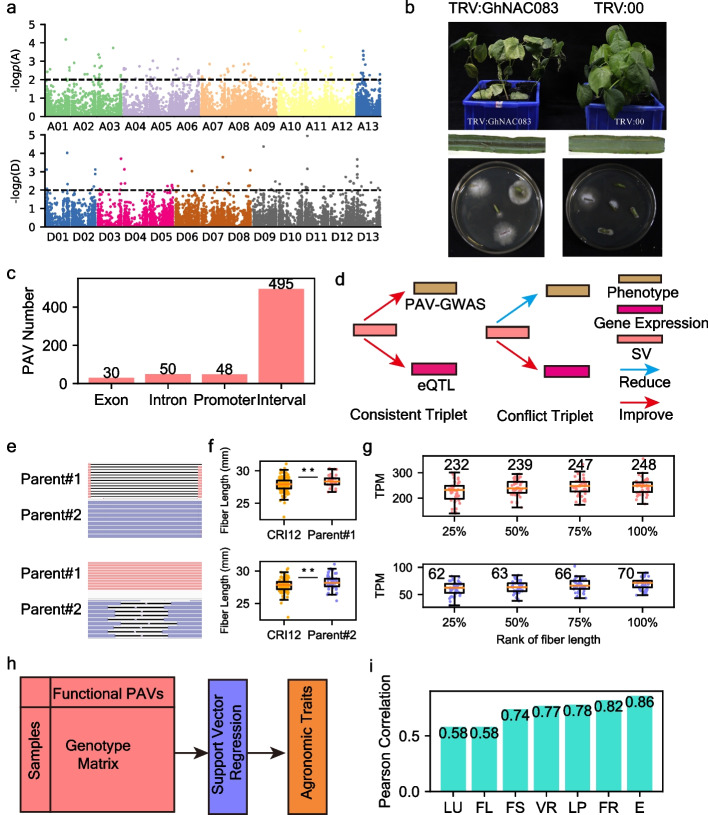
PAV-GWAS for agronomic traits. **a** PAV-GWAS for *Verticillium wilt* resistance, and the threshold of *P* value was set as 0.01. **b** VIGS assay on *GhNAC083*. **c** Genomic annotation for trait-related PAVs. **d** Concept illustration for consistent and conflict SV-Gene expression-Phenotype triplets. **e** Display of Parent#1_DEL_2442 and Parent#2_DEL_5283. The alignment of long reads from Parent#1 and Parent#2 were displayed in IGV toolkit. **f** Fiber length divergence of 2 genotypes compared to CRI12-like genotypes in Wang_2017 cohort (*n* = 169). Boxplot center, median; bounds of box, lower quartile (Q1) and upper quartile (Q3); minima, Q1 − 1.5 (Q1–Q3); maxima, Q1 + 1.5 (Q1–Q3). The dots represent for original values. *P* values were calculated by *t*-test and “**” represent for *P* ≤ 0.01. **g** Gene expression of *GhKCR1A* and *GhKCR1D* in wang_2017 cohort (*n* = 169). *X*-axis was the rank of fiber length (from short to long; divided into quartiles). *Y*-axes are TPM (transcripts per million mapped reads) of *GhKCR1A* and *GhKCR1D*, respectively. The description of boxes is the same as that in **f**, and “***” represents for *P* ≤ 0.001 in *t*-test. **h** Illustration for prediction of agronomic trait based on functional variations by support vector regression. **i** Pearson correlation between predicted phenotype and real phenotype

Using the GPG of CRI12, three previous cohorts (Ma_2018, Wang_2017, and He_2021) were genotyped, and trait-related PAVs were detected through PAV-GWAS for 7 agronomic traits (Fig. 3a; Additional file 1: Table S9-S11; Additional file 2: Fig. S2-S5). After applying strict filters to the detected trait-related PAVs (“Methods”), a functional PAV pool consisting of 623 variations was created (Additional file 1: Table S12). Within this PAV pool, we annotated 131 genes whose exons, introns, and promoters were directly influenced by PAVs (Additional file 1: Table S13). Of these 131 genes, 48 were related to resistance against *Verticillium wilt* (VW), and only 25 had transcriptional activity during *Verticillium dahliae* infections (Additional file 1: Table S14). We focused on *GhNAC083*, a gene previously reported as a repressor for xylem vessel formation in *Arabidopsis thaliana*, due to its opposite transcription patterns between VW-susceptible and VW-resistant accessions (Additional file 1: Table S15) [22]. As *NAC083* is involved in both biotic and abiotic stress responses, we inferred that it may also play an important role in cotton’s VW resistance [23]. In addition, *GhNAC083*-silenced plants are more susceptible to *Verticillium dahliae* infection compared to the wild type, indicating that *GhNAC083* could enhance VW resistance and be utilized in cotton breeding (Fig. 3b).

In the functional PAV pool, we discovered that 495 PAVs were located in the interval region, which was typically ignored in previous GWAS analyses, limiting the further utilization of elite genotypes (Fig. 3c) [14–16, 24, 25]. However, by utilizing an expression array with a population scale (Wang_2017 and He_2021 cohorts, *n* = 314 in total), we were able to link these functional non-coding PAVs to genes through eQTL analysis (Additional file 1: Table S10-S11) [15, 26]. Based on the effect of functional PAVs on agronomic traits and gene expression (positively or negatively associated), we classified all 2306 identified SV-Gene-Phenotype (SGP) triplets into consistent and conflict categories (Fig. 3d; Additional file 1: Table S16). Genes within consistent SGP triplets had higher expression with the gradual improvement of fiber quality, while those within conflict triplets showed a decline in gene expression as fiber quality improved (Additional file 2: Fig S6-S7). In the Wang_2017 cohort, we noted that enrichment of genes related to fatty acid metabolism (ko01212) among both consistent and conflict SGP triplets (Additional file 2: Fig. S8). The fatty acid metabolism pathway contained six genes including three genes each from the consistent and conflict SGP triplets (Additional file 1: Table S17). In the two consistent SGP triplets (Parent#1_DEL_2442-GhKCR1A-FL and Parent#2_DEL_5283-GhKCR1D-FL), the transcription abundance of *GhKCR1* increased from 1 day post anthesis (DPA) to 8 DPA, indicating its essential role in fiber elongation. Two different elite variations from Parent#1 and Parent#2 linked to homologs of *GhKCR1* in the A and D sub-genomes, respectively (Fig. 3e, f). The number of cultivars that contained these two elite variations increased with the improvement of fiber length (Additional file 2: Fig. S9). We noticed that *GhKCR1A* in long fiber cultivars (rank of fiber length at 50–75% and 75–100%) exhibited higher expression levels than short fiber cultivars (rank of fiber length at 0–25%) with both 6.5% increase (*P* values were 0.02 and 0.01, respectively) (Fig. 3g). *GhKCR1D* had similar transcription patterns with *GhKCR1A*, and long fiber cultivars (rank of fiber length at 50–75% and 75–100%) exhibited 6.5 and 13% increase in expression level (*P* values were 0.035 and 0.0025, respectively) compared to short fiber cultivars (rank of fiber length at 0–25%), respectively. This result shows that higher expression level of *GhKCR1*s is related with longer fiber length in a population scale. Interestingly, the expression of *GhKCR1* in the A sub-genome was higher than that in the D sub-genome, indicating asymmetric selection on *GhKCR1*s from different sub-genomes (Fig. 3g). *GhKCR1* encodes very-long-chain 3-oxoacyl-CoA reductase, responsible for the first reduction step in synthesis of very-long-chain fatty acids (VLCFAs) which is important for fiber elongation [27–29]. Based on gene annotation and the performance of the two SGP triplets in the population, we concluded that *GhKCR1*s identified from the above two SGP triplets could be used as candidate genes for improving fiber length. To examine the effects of the trait-related PAVs identified in this work, we constructed a set of support vector regression (SVR) models to predict agronomic traits based on genotype data (Fig. 3h). The Pearson correlation coefficient between predicted values and true values of multiple traits ranged from 0.58 to 0.85 (*P* values range from 1.09e − 52 to 1.38e − 129). These results show that except for fiber uniformity and fiber length, the other agronomic traits could be predicted reliably by models constructed based on functional PAVs (Fig. 3i).

Allelic variations were detected based on an accurate PAV map (482 pairs), and their effects were evaluated using the results of PAV-GWAS (Additional file 1: Table S18). We focused on a pair of non-coding allelic deletions, Xinluzhong7_DEL_3213-Parent#2 _DEL_2661 in the Wang_2017 cohort. On this locus, Parent#2-like genotypes had significantly higher fiber uniformity than Xinluzhng7-like genotypes (*P* = 0.002) (Additional file 2: Fig. S10a and S10b). However, compared to CRI12-like genotypes, both Parent#2-like and Xinluzhong7-like genotypes did not reach the significant divergence threshold in fiber uniformity (*P* = 0.07 and *P* = 0.3 for Parent#2-like and Xinluzhong7-like genotypes, respectively), resulting in their dismissal according to the threshold of PAV-GWAS described in the “Methods” (Additional file 2: Fig. S10a and S10b). Furthermore, we discovered 39 and 7 genes linked to Parent#2_DEL_2661 and Xinluzhong7_DEL_3213, respectively, from eQTL results, indicating that allelic variation might regulate different gene networks and lead to phenotype divergence (Additional file 1: Table S19; Additional file 2: Fig S10c).

### Low hereditary stability for functional segments in backbone cultivar

**Fig. 4:**
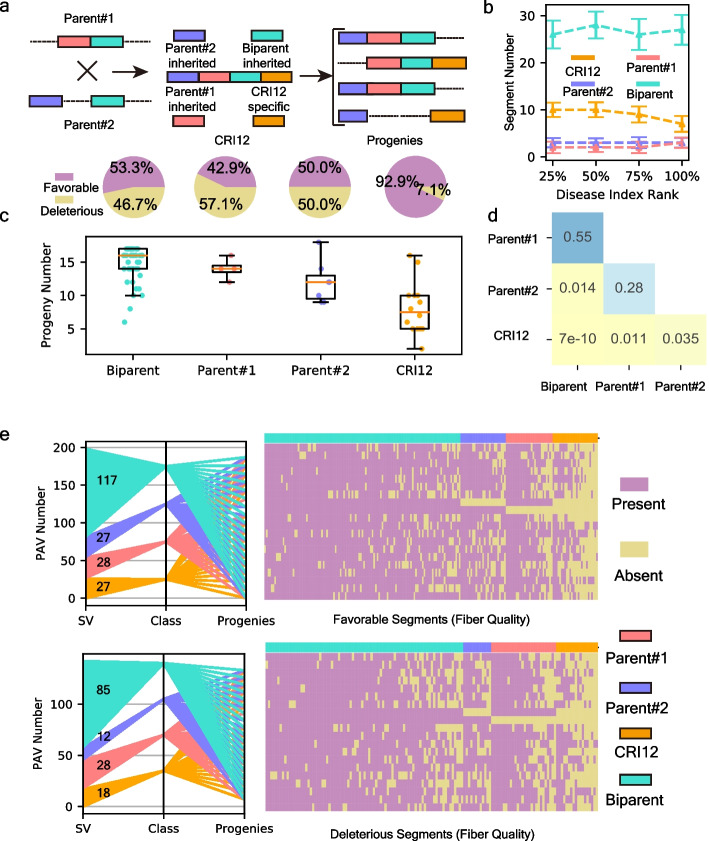
Genetic patterns of functional segments in CRI12 pedigree. **a** Conceptual illustration for segments of 4 categories. Parent#1-inhrited segments in CRI12 were inherited from Parent#1, presenting a structural variation in Parent#2. Parent#2-inherited segments in CRI12 were inherited from Parent#2, presenting a structural variation in Parent#1. CRI12-specific segments were those created by CRI12, presenting a structural variation in both Parent#1 and Parent#2. Biparent segments were those both present in CRI12’s 2 parents, only presenting a structural variation in progenies. **b** Relationship between *Fusarium wilt* resistance and functional segments of 4 categories in cultivars (Wang_2017 cohort, *n* = 208). *X*-axis represents for rank of disease index (from low to high; divided into quartiles) and *Y*-axis represents for segment number in cultivars from each quartile. Orange lines were CRI12-specific segments. Red lines were Parent#1-inherited segments. Blue lines were Parent#2-inherited segments, and turquoise lines were Biparent segments. The dots represent for the median values of segments in cultivars from each quartile. **c** Top 4 pieplots represent for favorable/deleterious ratio of 4 segment categories. The bottom boxplot was the hereditary stability evaluation of *Fusarium wilt*-resistant segments. *X*-axis represents 4 segment categories and *y*-axis represents the segment number retained in 18 progenies. Boxplot center, median; bounds of box, lower quartile (Q1) and upper quartile (Q3); minima, Q1 − 1.5 (Q1–Q3); maxima, Q1 + 1.5 (Q1–Q3). The dots represent for original values. **d** *P* value of *t*-tests for retained segments of 4 categories. **e** Distribution of fiber-quality-related segments in pedigree. The left parallel coordinate plots displayed segment number of 4 categories. SV axis represents for variations. Class axis represents for 4 segment categories which have been annotated in figure legends. Progenies axis represents for 18 progenies. Right heatmap were detailed presence of each functional segments in 18 progenies. *X*-axis represents for segments and *y*-axis represents for 18 progenies which are consistent with the progenies axis in left parallel coordinate plots. The top parallel coordinate-heatmap illustrates distribution of favorable fiber quality-related segments, while the bottom plot set illustrates deleterious fiber quality-related segments

The functional PAV pool of the CRI12 pedigree revealed that valuable variations were generated during its formation. Taking the phenotype effects into consideration, we defined a deleterious/favorable segment in CRI12 genome if the variation in alternative genome happened on corresponding locus could improve/reduce agronomic traits (Additional file 2: Fig. S11). Each functional variation in the pedigree represents either a favorable or deleterious segment in CRI12, and we classified all CRI12 segments into four categories such as Parent#1-inherited, Parent#2-inherited, CRI12-specific, and Biparental, based on the segment source (Fig. 4a). The source of Biparental segments in CRI12 is unclear due to the lack of genomic information about the parents of Parent#1 and Parent#2. Given that the Biparental segments were present in both Parent#1 and Parent#2, we concluded that they were likely inherited by CRI12’s parents from progenitor cultivars. The present/absent information regarding CRI12 segments among pedigree members enabled us to characterize the genetic patterns of the backbone cultivar in cultivar-driven strategy.

According to the results of PAV-GWAS on *Fusarium wilt* resistance (FR), 70 FR-related segments were identified. Among these 70 segments, 4, 7, 14, and 45 of them were assigned as Parent#1-inherited, Parent#2-inherited, CRI12-specific, and Biparental segments, respectively (Additional file 1: Table S20). We evaluated the effects of 70 FR-related segments belong to four categories in the Wang_2017 cohort (Fig. 4b). The cultivars with more CRI12-specific segments exhibited higher resistance to *Fusarium wilt* (lower disease index), indicating the positive effects of CRI12 in pathogen resistance. The ratio of favorable to deleterious segments in the CRI12-specific category reached 92.9% (13 favorable and 1 deleterious), while the ratios in the other three categories ranged from 42.9 to 53.3% (Fig. 4c). These results indicated that, compared to its parents, CRI12 generated more favorable FR-related segments, contributing to its pathogen resistance. However, we also found that these favorable CRI12-specific segments were less frequent in 18 progenies compared to segments inherited from its two parents and ancestral cultivars (*P* values ranged from 0.01 to 7e − 10), indicating low hereditary stability of CRI12-specific segments (Fig. 4c, d). The nature of the cultivar-driven strategy is to pass elite genomic segments from the backbone cultivar to its progenies. Our results showed the high ratio of favorable CRI12-specific segments, proving that CRI12 is competent to be a backbone cultivar, while their low hereditary stability suggests that they may be difficult to utilize adequately in a cultivar-driven strategy.

Among the 342 segments that influence fiber quality (FL, FS, FU, and FE), 199 were favorable while the remaining 143 were deleterious (Fig. 4e; Additional file 1: Table S21). The ratio of the four categories did not show any imbalance between the favorable and deleterious segments (*P* values range from 0.9 to 0.13, chi-square test), implying that the genomic resource for cotton fiber quality improvement in CRI12 pedigree is diverse (Fig. 4e; Additional file 1: Table S22). Similar to genetic pattern of 70 FR-related segments, both favorable and deleterious CRI12-specific segments presented low hereditary stability (*P* value ranged from 0.0008 to 3.5e − 40) (Fig. 4e; Additional file 2: Fig. S12). Moreover, we found that fiber quality-related biparental segments (both favorable and deleterious) were more stable in heredity than segments of the other three categories (*P* value were from 0.0012 to 3.5e − 40) (Fig. 4e; Additional file 2: Fig. S12). In the genetic trajectory of biparent segments in pedigree formation, we inferred that at least three rounds of selection for hereditary stability had occurred (from progenitor cultivars to Parent#1|Parent#2, from Parent#1|Parent#2 to CRI12, and from CRI12 to progenies). We concluded that only segments of high hereditary stability could be retained in progenies. This result showed that a cultivar-driven strategy could fix favorable segments of biparental category. However, deleterious segments from progenitor cultivars were fixed simultaneously during pedigree formation, and the accumulation of these highly stable segments could narrow the genetic background of the pedigree, damaging the potential of modern cultivars in cotton breeding.

### Geographically specific sub-groups in valuable segments

Cultivars in the CRI12 pedigree have been widely planted in China from the Northwest (Xinjiang Province) to the South (Hubei Province), suggesting the spread of favorable segments in CRI12 across different regions. To cluster functional segments into geographically specific sub-groups (SGs), we used the non-negative matrix factorization (NMF), an algorithm that has previously been used to detect genomic signatures [30].

**Fig. 5:**
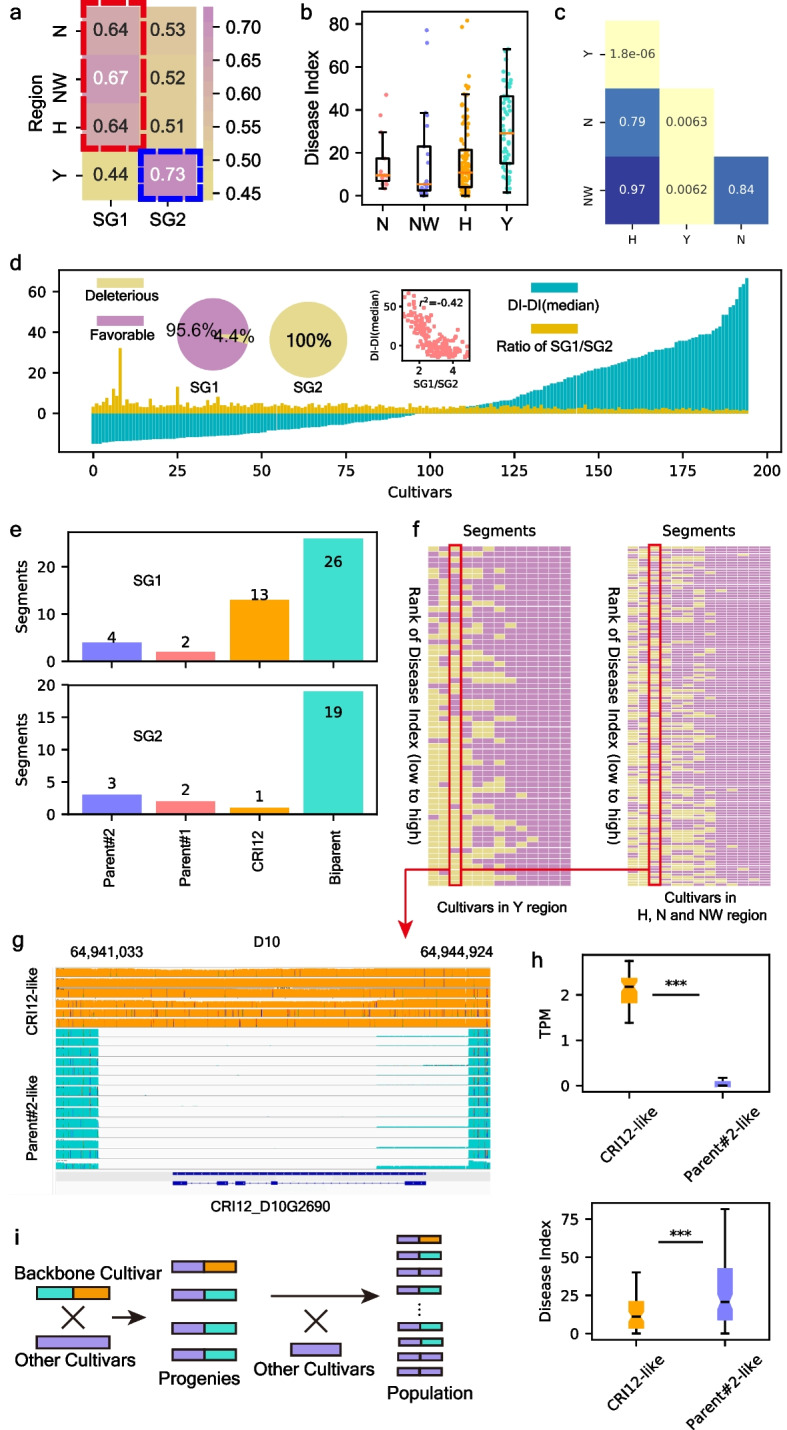
Geographical sub-groups in functional segments. **a** Cluster result of cultivars from 4 regions. The value in heatmap is the median feature-sample score of cultivars from each region. Four regions were clustered into two clades (red and blue dashed lines represent regions where segments belong to sub-group1 and sub-group2 enriched, respectively) by hierarchical method. **b** Disease index of cultivars from 4 regions in Wang_2017 cohort (*n* = 195). **c** *t*-test for disease index of cultivars from every 2 regions. **d** The left pieplots represented for ratio of favorable/deleterious segments in SG1 and SG2. The middle scatter plot was the Pearson correlationship between SG1/SG2 segments ratio and disease index (the disease index was normalized by median disease index). The yellow barplot was the SG1/SG2 segment ratio for each cultivar, while the blue barplot was the disease index for each cultivar normalized by median disease index in Wang_2017 cohort (*n* = 195). **e** Statistic about number of segments of 4 categories in SG1 and SG2. **f** The present/absent information of CRI12-specific segments in cultivars from Yangtze River region and the other 3 regions (Yellow River region, Northwestern region, early cotton producing region). **g** Illustration for deletions of *GhKHCP* in CRI12 pedigree. **h** The top boxplot was the transcription abundance of CRI12-like and Parent#2-like (with *GhKHCP* deletion) genotypes in Wang_2017 cohort (*n* = 195). The bottom boxplot was the disease index of 2 genotypes (Wang_2017 cohort; *n* = 195). *P* value was calculated by *t*-test and “***” represents for *P* ≤ 0.001. Boxplot center, median; bounds of box, lower quartile (Q1) and upper quartile (Q3); minima, Q1 − 1.5 (Q1–Q3); maxima, Q1 + 1.5 (Q1–Q3). **i** The conceptual illustration for bottleneck effect in cultivar-driven strategy. Orange segments represent for segments with low hereditary stability

To perform NMF analysis in the Wang_2017 cohort (195 cultivars retained after trimming foreign-introduced cultivars), we generated a present/absent matrix of 70 FR-related CRI12 segments, and two SGs were classified according to the feature-marker matrix (“Methods”; Fig. 5a; Additional file 2: FigS13a). The 195 retained cultivars were from four regions: the Yellow River region (H, 99 cultivars), the Yangtze River region (Y, 66 cultivars), the Northwest region (NW, 21 cultivars), and the early cotton producing region (N, 13 cultivars) (Additional file 1: Table S23). We noticed that segments in SG1 (45 segments) were preferentially present in cultivars from the N, NW, and H regions, while SG2 (25 segments) was preferentially present in cultivars from the Y region (Fig. 5a; Additional file 1: Table S24). The *Fusarium wilt* resistance of cultivars from the H, NW, and N regions was higher than that of cultivars from the Y region (*P* value was from 0.006 to 1.8e − 6), and among the three regions, the *Fusarium wilt* resistance of cultivars did not show any divergence (*P* value was from 0.79 to 0.97) (Fig. 5b, c). According to the results of the effect evaluation, we found that 95.6% of the segments in SG1 were favorable, while all segments in SG2 were deleterious (Fig. 5d). For each sample, the ratio of SG1 segments to SG2 segments was negatively related to the disease index, indicating that the ratio of SG1/SG2 segments is related to a cultivar’s *Fusarium wilt* resistance (Fig. 5d). Among the four categories, only CRI12-specific segments showed a significant divergent ratio between the two SGs (*P* = 0.01, chi-square test), and all favorable CRI12-specific segments belonged to SG1 (Fig. 5e). However, regardless of SG1 preference (those in the Y region) or SG2 preference (those in the H, N, and NW regions), several CRI12-specific segments were severely absent, indicating inadequate utilization of elite genomic resources (Fig. 5f). In these severely absent segments, a segment (D10: 64,941,033–64,944,924) contained a new gene, *CRI12_D10G2690* (Fig. 5g), which encodes a KH domain-containing protein (*GhKHCP*) which is involved in RNA-binding activities. A KH domain protein was shown to enhance the plant immune response in apple [31]. Cultivars without this segment showed a natural knock-out for *GhKHCP* (*P* = 0) and a significant increase in *Fusarium wilt* susceptibility (*P* = 0.0004) (Fig. 5h).

This segment contained *GhKHCP* only appeared in 18 and 43 cultivars from the Y and H regions, respectively (Additional file 2: Fig. S14). Correspondingly, there were only 6 progenies that inherited this CRI12-specific segment, showing its low hereditary stability (Fig. 5g). We calculated the present frequency of the 13,138 segments in the pedigree and large population (733 accessions) (Additional file 1: Table S26). Segments retained by less than 6 pedigree members had lower present frequencies in the large population than those retained by more than 12 pedigree members (*P* = 6.7e − 14), and we inferred that the “bottleneck effect” had occurred on these segments (Additional file 2: Fig. S15). The cultivar-driven strategy is widely applied in modern cotton breeding, in which cultivars from various pedigrees are utilized as elite gene donors. Thus, favorable segments with low hereditary stability are hard to spread widely among cotton germplasm (Fig. 5i).

We also performed NMF analysis on 59 fiber length-related segments from the Wang_2017 cohorts which were divided into two SGs: 29 segments in SG1 and 30 segments in SG2 and these classified cultivars into two clades (Additional file 1: Table S27; Additional file 2: Fig. S13b; Additional file 2: Fig. S16a). Clade I contained the cultivars from the N and NW regions, while clade II had the cultivars from the H and Y regions. Furthermore, we found that there were only 2 and 3 genes directly influenced by segments in SG1 and SG2, respectively (Additional file 1: Table S28). *CRI12_A09G0106* in SG2, which encodes an ISWI chromatin-remodeling complex ATPase, was identified because of an insertion in its last exon by Ekangmian10. Cultivars with this insertion had shorter fiber length (*P* = 0.007) and a lower expression level of *CRI12_A09G0106* (*P* = 1.33e − 5) (Additional file 2: Fig. S16b). ISWI members have been shown to be essential for the formation of heat stress memory in *Arabidopsis thaliana* [32]. In cotton, we also found that *CRI12_A09G0106* had divergent transcriptional patterns under heat and cold stresses (Additional file 2: Fig. S16c). Interestingly, the transcriptional abundance of *CRI12_A09G0106* increased during heat stress, while its expression was repressed in long fiber cultivars, implying its contradictory role in the heat stress response and fiber elongation.

### Fingerprint segments in CRI12 pedigree

**Fig. 6:**
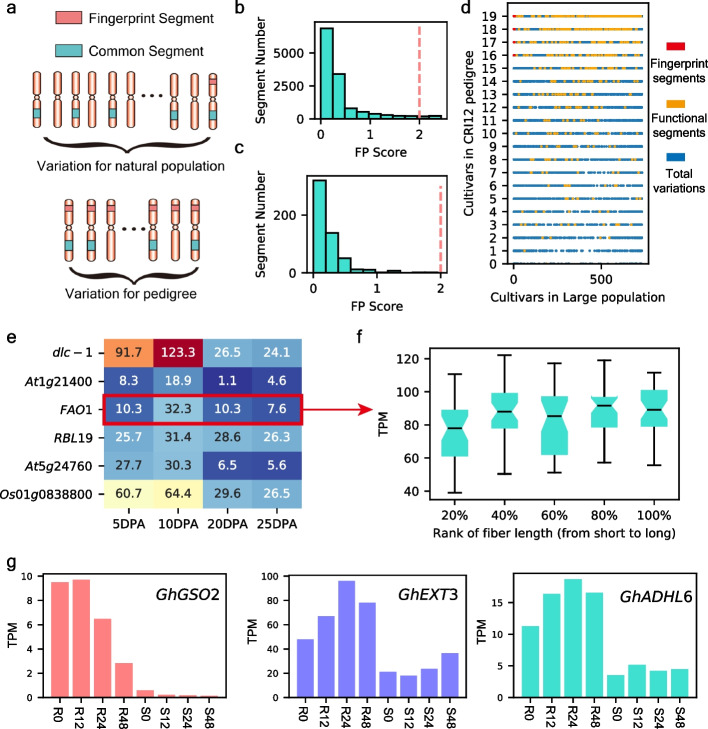
Fingerprint segments in CRI12 pedigree. **a** Conceptual illustration for pedigree fingerprint segments.** b** Fingerprint score of 13,138 segments. **c** Fingerprint score of functional segments. **d** Presence/absence of 13,138 segments in CRI12 pedigree and 733 cultivars. **e** Transcription abundance of fiber length-related candidate genes influenced by fingerprint segments. **f** Transcription abundance of *GhFAO1* in cultivars with various fiber length. TPM (transcripts per million mapped reads). Boxplot center, median; bounds of box, lower quartile (Q1) and upper quartile (Q3); minima, Q1 − 1.5 (Q1–Q3); maxima, Q1 + 1.5 (Q1–Q3). **g** Transcription abundance of 3 *Verticillium wilt*-resistant genes. For *X*-axis, R represents for resistant accession and S represents for susceptible accession, while numbers represent hours after pathogen infection

In the above results regarding “bottleneck effects,” we have identified that segments with low hereditary stability always had low present frequencies in large populations. However, we also found several segments that were retained by most pedigree members but were also rare in the large population. We assigned these pedigree-locked segments as “pedigree FingerPrint Segments” (FPS) (Fig. 6a). To detect the FPS quantitatively, we applied TF-IDF (term frequency–inverse document frequency) algorithm (“Methods”) to calculate the fingerprint score of each segment (Additional file 1: Table S29). Finally, we identified 367 FPS by setting the threshold of the fingerprint score as 2, in which no functional segments were included (Fig. 6b and c; Additional file 1: Table S30). We checked the pedigree-population distribution of both FPS and functional segments, indicating that the TF-IDF algorithm had accurately detected the FPS (Fig. 6d). Most functional segments were broadly introduced to cultivars in the large population, while fingerprint segments were restricted to the pedigree members, presenting a pedigree-lock on them (Fig. 6d). The genomic annotation showed that 265 FPS were in the genomic interval region, hindering the comprehensive interpretation of these pedigree-locked segments (Additional file 1: Table S30). Thus, we focused on those influencing genes directly (overlapped with exons, introns, and promoters), and a total of 126 genes were gained. The KEGG enrichment in 126 genes showed that there were no significant enrichment items, indicating that FPS-influenced genes had no pathway preference and these genes affect multiple traits.

We used a temporal fiber development transcriptome to select six candidate genes that could potentially contribute in fiber elongation (highest expression level on 10 DPA) (Fig. 6e). Among the six candidate genes, only *GhFAO1* was found to be related to fiber length on a population scale (Fig. 6f; Additional file 1: Table S31). *GhFAO1* encoded a long-chain fatty alcohol oxidase that can oxidize long-chain fatty alcohol into long-chain fatty aldehyde, which is the precursor of long-chain fatty acid [33]. We inferred that *GhFAO1* is an important gene controlling the upstream reactions of long-chain fatty acid synthesis. We also identified three *Verticillium* wilt-resistant genes (larger than twofold change in mean expression level of resistant and susceptible plants), *GhGSO2*, *GhEXT3*, and *GhADHL6*, using transcriptome data (*P* value ranging from 0.01 to 0.0004) (Fig. 6g). *GSO2* encoded an LRR receptor-like serine/threonine-protein kinase that could help to construct the Casparian strip diffusion barrier, trapping pathogens [34]. *EXT3* is a structural component that strengthens the primary cell wall to protect the cell from pathogens [34, 35]. Combining with the functional annotation and higher expression levels in resistant accessions, these three genes were referred as potential gene resources for improving *Verticillium wilt* resistance. The above results showed that FPS, although was locked in the pedigree, still contained elite gene resources that could only be detected by comparing genomic landscapes between the pedigree and natural population.

## Discussion

Backbone cultivars, which possess elite gene resources, have played a crucial role in the history of cotton breeding [11, 25, 36]. Despite numerous genome-wide association studies identifying favorable loci, the utilization of elite genomic segments from backbone cultivars still remains limited in practical cotton breeding [7, 14–16, 24, 25]. Therefore, it is imperative to integrate pedigree genomic analysis into cotton breeding practices to fully leverage elite genomic segments offered by backbone cultivars. We constructed GPG (Graphical Pedigree Genome) of CRI12 to capture genomic variations in CRI12 pedigree. CRI12’s GPG provides a platform for gene clone, genome-wide association studies, and pedigree genomic analysis, discovering low hereditary stability and geographical grouping of elite segments in backbone cultivar. In addition, we proposed a new concept called “pedigree fingerprint segments” to identify potential elite segments that are still confined within pedigree.

Graphical genomes with a large number of SVs have exhibited strong utility in many other crops, including cotton [11, 37–39]. However, the identification of SVs and the construction of graphical genomes require a set of high-quality assembled genomes [40]. The detection of SVs using short reads results in high false positive rate, and genome-based SV detection is impractical for cohorts with hundreds of individuals [41, 42]. In this study, we introduce a cost-effective strategy for dependable graphical genome construction using long reads with moderate depth. The utility of this strategy for genetic analysis in cohorts is demonstrated by the representativeness of PAVs in GPG. Previous PAV-GWAS based on cotton pan-genomes provided valuable PAVs associated with fiber yield and quality, allowing identification of causal genes [11, 25]. Through the utilization of GPG, we successfully identified 623 functional genomic segments associated with fiber yield, fiber quality, and pathogen resistance. These segments have the potential to serve as markers for genome selection, with the exception of those influencing two fiber quality traits, fiber uniformity, and fiber length. It is worth noting that for these two traits, the predicted values showed a weak Pearson correlation with the actual traits. Because GPG only considers variations in pedigree, it may overlook variations related to fiber uniformity and fiber length compared to a cotton pan-genome including 182,593 SVs [11].

The limited hereditary stability of elite genomic segments in backbone cultivars poses a restriction on the application of a cultivar-driven breeding strategy. This highlights the need for a shift in breeding strategy from cultivar-driven to gene-driven approaches in modern crop breeding. Genetic analysis based on large genomic variations have constructed functional genomic segment pools which not only contained elite genes and cis-eQTLs but also valuable trans-eQTLs [10, 11, 25]. The development of plant genome editors has facilitated the precise insertion of large sequences, enabling the manipulation of elite genomic segments [43]. This is particularly advantageous for segments that contain multiple genes or are located within genomic interval regions. After introducing a set of elite segments into cultivars using genome editors, the optimal combination of genomic segments can be identified through genome selection based on GPG. Thus, we propose a 4G cotton breeding strategy, GPG construction-Genomic analysis-Genome editing-Genome selection, to make full use of elite genomic segments in backbone cultivars. The strategy mainly depends on the initial step, GPG construction. This step requires the characterization of the genomic landscape of multiple pedigrees to build an integrated pedigree genomic resource map capturing functional segments and pedigree fingerprint segments present in all backbone cultivars.

## Conclusions

The PAV-GWAS based on GPG had detected a set of favorable segments in CRI12 pedigree which could be used in cotton molecular breeding. Revealing genetic basis of cultivar-driven strategy’s restriction provides theorical support for breeding strategy shift. In the future, we hope that genomic landscapes of more cotton pedigrees are characterized to construct an entire pedigree genomic resource map capturing elite segments from all cotton backbone cultivars.

## Methods

### Plant material

*G. hirsutum. cv* CRI12 and its 20 pedigree members (2 parents and 18 progenies) were cultivated in the field of Institute of Cotton Research, Chinese Academy of Agricultural Sciences, Anyang, Henan, China. For each pedigree member, three leaves (after the third leaf spread) from five plants were harvested and frozen immediately in liquid nitrogen. Six tissues from CRI12 plants including stem, root, leaf, petal, ovule (0 DPA), and fiber (1 DPA) were collected to perform transcriptome analysis for genome annotation.

### Genomic DNA extraction and genome sequencing

Total genomic DNA from collected leaves was extracted for Nanopore long-read sequencing using a Nanopore PromethION 48 sequencer. Genomic DNA was fragmented by Covaris g-TUBE. Subsequently, DNA repair and adapter ligation were performed. Finally, DNA fragments longer than 3 kb were collected for library construction..

For Illumina short-read sequencing, gel electrophoresis was used to detect degradation and contamination of DNA on 1% agarose gel. AMPure XP system (Beckman Coulter, Beverly, USA) was used to purify DNA, and the 3’ end of the DNA fragment was adenylated and then linked with an adaptor for hybridization. Electrophoresis was used to select DNA fragments with proper length in PCR assays which were subsequently purified for library construction. The products in library were clustered and sequenced on Illumina Hiseq platform to generate reads with the length of 150 bp.

To perform Hi-C sequencing, DNA extracted from young leaves of CRI12 was cross-linked and then digested with a restriction enzyme, DpnII. The fragments digested by the enzyme were biotinylated and the read pairs with physical interaction will be ligated to each other which were enriched for library construction and were sequenced by Illumina Hiseq platform.

### De novo assembly of CRI12 genome

About 280 Gb raw data generated by Nanopore long-read sequencing was used to perform draft genome assembly. The quality control of Nanopore reads was performed by Nanoplot (v1.40.0). Filtered Nanopore long reads were assembled by NextDenovo (https://github.com/Nextomics/NextDenovo). The result of NextDenovo was transferred to SmartDenovo (https://github.com/ruanjue/smartdenovo) for tandem assembly. Racon was applied to polish the tandem assembly by NextDenovo and SmartDenovo for three times [44]. Subsequently, Illumina short reads with genomic coverage of 60-fold were filtered by Fastp and utilized to correct sequencing errors within Nanopore long reads by Pilon [45, 46]. The contigs polished by Illumina short reads were regarded as draft genome and assembled into chromosomes by Hi-C reads. Hi-C reads were aligned to draft genome by Bowtie2 (v.2.4.5) [47]. Juicer pipeline (https://github.com/aidenlab/juicer) was used to analyze alignment results and to generate an interaction matrix [48]. The interaction matrix was treated by 3d-dna pipeline (https://github.com/theaidenlab/3d-dna) to perform chromosome scaffolding [49]. The misassemblies within hanged chromosomes were corrected manually by Juicerbox (https://github.com/aidenlab/Juicebox/wiki/Download).

### Genome annotation of CRI12

We employed three strategies including de novo prediction, protein homology prediction, and transcript-based prediction to annotate genes in CRI12 genome.

For de novo prediction, four software, Augustus (https://github.com/Gaius-Augustus/Augustus), GlimmerHMM (http://ccb.jhu.edu/software/glimmerhmm/), Geneid (https://genome.crg.es/software/geneid/), and Genscan (http://genes.mit.edu/burgelab/software.html), were applied [50–53]. Protein coding sequences from *G. hirsutum* (http://cotton.hzau.edu.cn/EN/download.php), *G. barbadense* (http://cotton.hzau.edu.cn/EN/download.php), *G. arboreum* (http://bioinfo.ayit.edu.cn/downloads/), and *G. raimondii* (https://phytozome.jgi.doe.gov/pz/portal.html#!bulk?org=Org_Graimondii) were collected for homology prediction. Transcripts from six CRI12 tissues including roots, stems, leaves, petals, ovules on 0 DPA, and fibers on 1 DPA were used for transcript-based prediction. We used EvidenceModeler (https://github.com/EVidenceModeler/EVidenceModeler/releases) to integrated gene models generated by above three strategies [54]. The functions of these predicted protein sequences were annotated by interproscan (v.5.51–85.0) [55]. Repeat regions were gained through the combination of de novo prediction and homology prediction by RepeatMasker (v.4.1.0) [56, 57].

### Phenotype data collection of pedigree varieties

The phenotype data of CRI12 pedigree varieties were collected from different locations (8 locations for fiber yield and 9 locations for fiber quality) in 2 years (2019, 2020), and each natural environment had 3 biological replicates. Lint percentage (LP) was calculated based on harvested fibers from 30 balls. The fiber quality traits including half mean length (FL), fiber uniformity (FU), fiber strength (FS), and elongation (E) were measured. Fiber quality trait was evaluated by a high-volume instrument (HFT9000) at the Cotton Quality Testing Center in Institute of Cotton Research, Chinese Academy of Agricultural Science (CRI, CAAS). For *Verticillium wilt* and *Fusarium wilt* resistance, GB/T22101.5–2009 was used to calculate disease index. Three replicates were planted (6 pots were used for each replication and 4 plants were planted in each pot) in a greenhouse to observe phenotype after infection. The phenotypic data was normalized by maximum value to perform hierarchical cluster.

### SV detection in CRI12 pedigree varieties

About 28 to 69 Gb raw data was generated for 20 pedigree members after ONT sequencing. Subsequently, long reads were aligned to the CRI12 genome by NGMLR (v0.2.7) with the parameter –x ONT after quality control by Nanoplot (v1.40.0) with default parameter [56, 57]. Based on alignment results, Sniffles Package (v1.0.12) was used to detect SVs which were longer than 20 bp [58]. Simultaneously, cuteSV (v1.0.9) was also applied to detect SVs (larger than 20 bp) from NGMLR results [59]. Only SVs from two SV sets (generated by Sniffles and cuteSV) had an over 50% reciprocal overlap were retained as final SVs. According to a reported strategy, SVs detected from each pedigree cultivar were merged into a non-redundant SV set [60]. The sequences of non-redundant SV set were extracted for transposon detection with RepeatMasker (v.4.1.0) [56, 57].

### GPG construction, PAV-GWAS, and eQTL analysis

Sequences of insertions and deletions (PAVs) in the non-redundant SV set were collected and integrated into CRI12 genome by vgtoolkit (v1.33.0) to build a graphical pedigree genome (GPG) [61]. Raw data of samples in 3 cohorts were downloaded according to accession provided by NCBI. The phenotype data of ma_419 was downloaded (http://cotton.hebau.edu.cn/). The Best Linear Unbiased Prediction (BLUP) value of phenotype data from 12 natural environments was gained through the R package lme4 (v1.1–7). The phenotype of samples in the other 2 cohorts is available in corresponding studies (Phenotype data of individuals in He_2021 is available at https://doi.org/10.1038/s41588-021-00844-9; while *Fusarium wilt* resistance of Wang_2017 is available at https://doi.org/10.1002/advs.202002723; fiber-related traits of Wang_2017 are available at https://doi.org/10.1038/ng.3807). Short reads of cultivars in 3 cohorts were aligned to GPG by vgtoolkit (v1.33.0) to generate the genotype matrix [61]. With the combination of phenotypic and genotypic matrices, we used EMMAX (http://csg.sph.umich.edu/kang/emmax/download/index.html) to perform PAV-GWAS to identified PAVs related to agronomic traits including VW resistance, FW resistance, fiber length, fiber strength, fiber uniformity, fiber elongation, and lint percentage.

Because of small marker number, markers’ − log_10_ (*P*) value is low. To select trait-related variations and avoid false positive results, we set 0.01 *P* value for marker selection, and phenotype divergence between reference and alternative genotypes was significant at *P* ≤ 0.05. Moreover, after phenotype-genotype reciprocal check, PAVs were filtered by its influence on gene expression in a population scale. Finally, functional PAV pool was built by phenotypic-gene expression filtering on significant PAVs from EMMAX results [62].

Transcriptome data of 314 samples of wang_2017 and He_2021 cohorts were collected from NCBI. We applied Hisat2-Stringtie pipeline to gain transcription abundance of each gene in CRI12 genome [63, 64]. Using gene expression and genotype matrix as phenotype and genotype file, respectively, we performed eQTL analysis by EMMAX [62].

### Virus induced gene silencing and *V. dahliae* inoculation

A specific fragment of *GhNAC083* in cotton cultivar CRI12 was amplified and inserted to a tobacco rattle virus vector (pTRV2). As a phenotype marker of the VIGS experiment, fragments of PDS were also inserted to pTRV2. pTRV2 and pTRV1 were transferred into *Agrobacterium tumefaciens* GV3101. The CRI12 plants were cultivated in illumination incubator with 16 h/8 h light/dark at 25℃. Plants inoculated with empty pTRV2 and pTRV1 were used as mock controls. The VIGS procedure was implemented according to the previously reported method [65]^66^. Two weeks after *Agrobacterium tumefaciens* inoculation, PDS-silenced plants turned to be bleach, and we inoculated the *GhNAC083*-silenced and control plants with 15 mL Vd076 spore suspension at about 1 × 10^7^ spores/mL after hurting the roots of a plant for each sample. Afterwards, we checked the phenotype of inoculated plants after 14 days after inoculation of *V. dahliae*, observing the splitted stems by a microscope. To check the colonization of the *V. dahliae* in the plants, we selected splitted stems from inoculated plants and cultured them on PDA agar medium for 3 days.

The front primer of *GhNAC083* for VIGS is GTAATGAGAGTAACA, while the reverse primer of *GhNAC083* is CCATGCCATTCCCTC.

### SV visualization

The visualization of SVs in CRI12 pedigree cultivars was performed by IGV (https://github.com/igvteam/igv) [66].

### Construction of phenotypic prediction models

For each agronomic trait, corresponding PAVs in functional PAV pool were selected as features in a support vector regression model which is built by scipy (v.0.12.0). The Pearson correlation between predicted and real phenotypes was calculated by pearsonr function in scipy.

### Non-negative matrix factorization analysis

We transferred PAV genotype matrix of 733 cultivars into segment retain matrix and applied NMF function in sklearn (v.1.1.3) package in python to perform NMF analysis. The segment retain matrix has shape (m, n) in which m presents for m segments and n presents for n cultivars. Segment_i,j_ = 1 means cultivar_j_ had segment_i_, otherwise, segment_i_ is absent in cultivar_j_. In NMF pipeline, the segment matrix S_m,n_ was factorized into 2 matrices, W_m,f_ (feature-marker matrix) and H_f,n_ (feature-sample matrix). The value of f is the compressed feature numbers in NMF (the number of SGs in this study). Feature number was set from 2 to 10 and evaluated by silhouette score calculated from results of KMeans (in sklearn package) cluster on matrix W_m,f_. W_m,f_ and H_f,n_ enable us to classify segments and samples into sub-groups, respectively. For segments, if max (value_m,1_, value_m,2_…value_m,f_) = value_m,f_ (value_i,j_ ∈ W_m,f_), we assigned segment_m_ as member in sub-group f, and value_m,f_ was assigned as feature-marker score. For samples, if max (value_1,n_, value_2,n_…value_f,n_) = value_f,n_ (value_i,j_ ∈ H_f,n_), we assigned that sub-group f was preferentially present in sample_n_, and value_f,n_ was assigned as feature-sample score.

### Identification of fingerprint segments

Segments in CRI12 which were widely inherited to progenies but absent in 3 cohorts were assigned as pedigree fingerprint segments (PFS). To identify PFS in CRI12 pedigree, we utilized tf-idf (term frequency–inverse document frequency) algorithm which was used to deal keyword extraction in natural language processing researches. In this study, term frequency of segment_i_ (tf_i_) was defined as number_progenies with segmenti_/20, in which 20 represented for 20 pedigree members we collected. While inverse document frequency of segment_i_ (idf_i_) was defined as lg(733/number_cultivars with segmenti_) in which number_cultivars with segmenti_ represented the number of cultivars with segment_i_ in 3 cohorts, and 733 represented the total cultivar number in 3 cohorts. Fingerprint score for segment_i_ was defined as tf_i_ × idf_i_, and segments whose fingerprint score larger than 2 was identified as fingerprint segments.

### Statistical analysis

All statistical analysis in this research was implemented by scipy package in python (v3.8).

### Supplementary Information

**Additional file 1: Table S1.** Summary Statistics about Nanopore Sequencing Reads. **Table S2.** Statistic About Illumina Short Reads for Genome assembly. **Table S3.** Statistic for Hi-C Reads from 2 Replicates. **Table S4.** Syntenic Ratios Compared with CRI12. **Table S5.** Phenotype data of CRI12 pedigree members. **Table S6.** The summary statistics about Nanopore long reads of 20 CRI12 pedigree members. **Table S7.** Genomic annotation for pedigree structural variations. **Table S8.** Transposon Annotation for each SV. **Table S9.** Basic information about 3 cohorts. **Table S10.** SRR accession number of 169 samples in Wang_2017 cohort. **Table S11.** SRR accession number of 145 samples in He_2021 cohort. **Table S12.** Statistic about functional PAVs. **Table S13.** Genomic annotation for functional PAVs. **Table S14.** Functional annotation for genes directly influenced by functional PAVs. **Table S15.** Transcription abundance of VW-related genes between resistant and susceptible accessions. **Table S16.** SV-Gene expression-Phenotype triplets underlying non-coding SVs. **Table S17.** 6 genes in fatty acid elongation pathways. **Table S18.** Allelic SVs in CRI12 pedigree. **Table S19.** Regulation networks formed by a pair of allelic deletions. **Table S20.** Category information about 70 Fusarium wilt resistance segments. **Table S21.** Category information about fiber-quality segments. **Table S22.** Chisquare test for fiber-quality segments. **Table S23.** Source of 195 samples in Wang_2017 cohort. **Table S24.** Sub-groups in Fusarium wilt resistance-related segments. **Table S25.** Chisquare test for 4 categories in 2 sub-groups divided from 70 FR segments. **Table S26.** Distribution of segments in CRI12 pedigree and population with 733 samples. **Table S27.** Sub-groups in Fiber-quality related segments. **Table S28.** Genomic annotation about fiber-quality related segments. **Table S29.** Fingerprint score of 13138 segments. **Table S30.** Genomic annotation for fingerprint segments in CRI12 pedigree. **Table S31.** t-test about fiber length of 5 sub-populations in Wang_2017 cohort.

**Additional file 2: Fig. S1.** Statistic about transposons in CRI12 pedigree PAVs. a. Pearson correlationship between number of transposon and PAV length in 4 genomic regions. b. Ratio of 26 tranposon categories in 4 genomic regions. The sum of all values in a column was 1. **Fig. S2.** GWAS for fiber-related traits in Ma_2018 cohort (*n*=419). a-e. Manhattan plot and Q-Q plot for lint percentage, fiber length, fiber strength, fiber uniform and fiber elongation. Threshold of *P*-value was set as 0.01 and all selected locus were furtherly filtered as described in Methods. **Fig. S3.** GWAS for fiber-related traits in Wang_2017 cohort (*n*=169). a-d. Manhattan plot and Q-Q plot for fiber length, fiber strength, fiber uniform and fiber elongation. Threshold of *P*-value was set as 0.01 and all selected locus were furtherly filtered as described in Methods. **Fig. S4. **GWAS for fiber-related traits in He_2021 cohort (*n*=145). a-c. Manhattan plot and Q-Q plot for lint percentage, fiber length, fiber strength and fiber elongation. Threshold of *P*-value was set as 0.01 and all selected locus were furtherly filtered as described in Methods. **Fig. S5.** GWAS for pathogen-resistance traits in Ma_2018 cohort (*n*=408) and Wang_2017 (*n*=208). a-b. Manhattan plot and Q-Q plot for Fusarium wilt resistance in Wang_2017 cohort (*n*=208). c. Q-Q plot for GWAS on Verticillium wilt resistance in Ma_2018 cohort (*n*=408). **Fig. S6.** Transcription abundance of genes from SGP triplets in Wang_2017 (*n*=169). a. Genes in consistent SGPs related to fiber strength, fiber uniform and fiber elongation. X-axis was the rank of agronomic trait (from low to high in quartiles). Y-axis was the TPM of genes in Wang_2017 cohort. b. Genes in conflict SGPs related to fiber strength, fiber uniform and fiber elongation. **Fig. S7. **Transcription abundance of genes from SGP triplets in He_2021 (*n*=145). a. Genes in consistent SGPs related to fiber length, fiber strength and fiber elongation. X-axis was the rank of agronomic trait (from low to high in quartiles). Y-axis was the TPM of genes in He_2021 cohort. b. Genes in conflict SGPs related to fiber length, fiber strength and fiber elongation. **Fig. S8.** Genes from SGP triplets about fiber length in Wang_2017 (*n*=169). a. Left boxplot was transcription abundance of genes in consistent SGPs related to fiber length. X-axis was the rank of fiber length (from short to long in quartiles). Right barplot was the KEGG result of genes in consistent SGPs in Wang_2017 cohort. b. Left boxplot was Transcription abundance of genes in conflict SGPs related to fiber length. X-axis was the rank of fiber length (from short to long in quartiles). Right barplot was the KEGG result of genes in conflict SGPs in Wang_2017 cohort. **Fig. S9.** Two elite deletions contained in Wang_2017 (*n*=169). Fiber length was sorted into quartiles from short to long. The total number of 2 deletions contained by cultivars in each quartile was counted. **Fig. S10.** Allelic fiber uniform-related SVs in CRI12 pedigree. a.Allelic deletions in CRI12 pedigree. Purple lines represent for Uganda4_DEL_2661; Orange lines represent for CRI12 genotypes and blue lines represent for Xinluzhong7 genotypes. b. Fiber uniform of 3 genotypes. c. Gene regulatory network of allelic deletions. **Fig. S11.** Illustration on relationship between variations in alternative genomes and segments in CRI12 genome. Variations in alternative genomes reducing agronomic trait are mapped to corresponding favorable segments in CRI12 genome (segment1 and segment4 in CRI12 improve trait). Variations in alternative genomes improving agronomic trait are mapped to corresponding deleterious segments in CRI12 genome (segment2 and segment3 in CRI12 reduce trait). **Fig. S12.** Hereditary stability of fiber quality-related segments in CRI12 pedigree. a. The boxplot of pedigree favorable segments of 4 categories. b. The boxplot of pedigree deleterious segments of 4 categories. c. t-test on hereditary stability among favorable segments of 4 categories. d. t-test on hereditary stability among deleterious segments of 4 categories. **Fig. S13.** Feature number in NMF analysis. a. Selection on number of features in 70 Fusarium wilt resistant segments. b. Selection on number of features in fiber length related segments. X-axis is the number of features in NMF analysis and Y-axis is the silhouette score of sample-feature matrix. **Fig. S14.** Number of cultivars containing *GhKHCP* from 4 regions. The orange bars are numbers of cultivars with *GhKHCP* from 4 regions, while turquoise bars are numbers of cultivars without *GhKHCP* from 4 regions. Cultivars without *GhKHCP* were enriched in Yangze River region (χ^2^ test). **Fig. S15.** Bottoleneck effect in CRI12 pedigree. Orange box is the present frequency of segment with low hereditary stability in 733 population. While, turquoise box is the present frequency of segment with high hereditary stability in 733 population (*P*=6.7e-14). **Fig. S16.** NMF result for fiber length-related segments. a. Cluster result of cultivars from 4 regions. The value in heatmap is the median feature-sample score of cultivars from each region, and 4 regions were clustered by hierarchical method. b. Fiber length of 2 genotypes (CRI12-like and Ekangmian10-like) and the transcription abundance of *CRI12_A09G0106* of 2 genotypes. c. The expression of *CRI12_A09G0106* after cold and heat treat in 1, 3, 6 and 12 hours.

**Additional file 3. **Review history.

## Data Availability

All data (ONT long reads, Illumina short reads, Hi-C data, and transcriptome data) used for CRI12 assembly is available at PRJNA1000640 [67]. ONT long reads for 20 CRI12 pedigree members are available at PRJNA1000641 [68]. Previous published data used in this paper: (i) Transcriptome data of Verticillium wilt infection was fetched in BioProject PRJNA593765 [69]. (ii) Transcriptome data of fiber development was fetched in SRA database with accession number SRR2917183- SRR2917197 and SRR1695191-SRR1695194 [70, 71]. (iii) Accession numbers of the genomic and transcriptome data from 3 cohorts were available in Supplementary table 10 and 11 [14–16, 26]. There were no scripts and software used other than those mentioned in the “Methods” section.
