# Supplementary material for: Cotton pedigree genome reveals restriction of cultivar-driven strategy in cotton breeding
Source: Genome Biol. 2023 Dec 8;24:282. doi: 10.1186/s13059-023-03124-3 (PMC10704732; doi:10.1186/s13059-023-03124-3)
Supplement: Supplementary file 2 — Additional file 2: Fig. S1. Statistic about transposons in CRI12 pedigree PAVs. a. Pearson correlationship between number of transposon and PAV length in 4 genomic regions. b. Ratio of 26 tranposon categories in 4 genomic regions. The sum of all values in a column was 1. Fig. S2. GWAS for fiber-related traits in Ma_2018 cohort (n=419). a-e. Manhattan plot and Q-Q plot for lint percentage, fiber length, fiber strength, fiber uniform and fiber elongation. Threshold of P-value was set as 0.01 and all selected locus were furtherly filtered as described in Methods. Fig. S3. GWAS for fiber-related traits in Wang_2017 cohort (n=169). a-d. Manhattan plot and Q-Q plot for fiber length, fiber strength, fiber uniform and fiber elongation. Threshold of P-value was set as 0.01 and all selected locus were furtherly filtered as described in Methods. Fig. S4. GWAS for fiber-related traits in He_2021 cohort (n=145). a-c. Manhattan plot and Q-Q plot for lint percentage, fiber length, fiber strength and fiber elongation. Threshold of P-value was set as 0.01 and all selected locus were furtherly filtered as described in Methods. Fig. S5. GWAS for pathogen-resistance traits in Ma_2018 cohort (n=408) and Wang_2017 (n=208). a-b. Manhattan plot and Q-Q plot for Fusarium wilt resistance in Wang_2017 cohort (n=208). c. Q-Q plot for GWAS on Verticillium wilt resistance in Ma_2018 cohort (n=408). Fig. S6. Transcription abundance of genes from SGP triplets in Wang_2017 (n=169). a. Genes in consistent SGPs related to fiber strength, fiber uniform and fiber elongation. X-axis was the rank of agronomic trait (from low to high in quartiles). Y-axis was the TPM of genes in Wang_2017 cohort. b. Genes in conflict SGPs related to fiber strength, fiber uniform and fiber elongation. Fig. S7. Transcription abundance of genes from SGP triplets in He_2021 (n=145). a. Genes in consistent SGPs related to fiber length, fiber strength and fiber elongation. X-axis was the rank of agronomic trait (from low to hig [file 13059_2023_3124_MOESM2_ESM.docx]

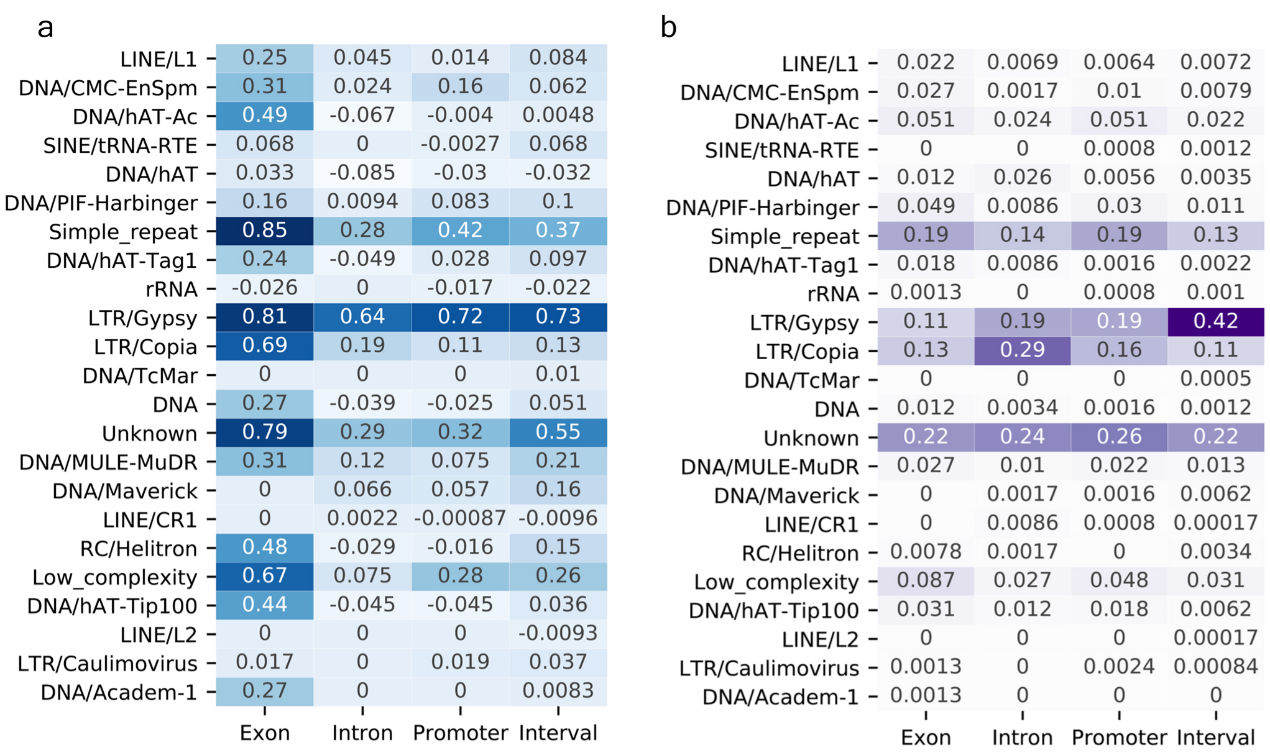


**Fig. S1** |Statistic about transposons in CRI12 pedigree PAVs.

**a**. Pearson correlationship between number of transposon and PAV length in 4 genomic regions.

**b**. Ratio of 26 tranposon categories in 4 genomic regions. The sum of all values in a column was 1.


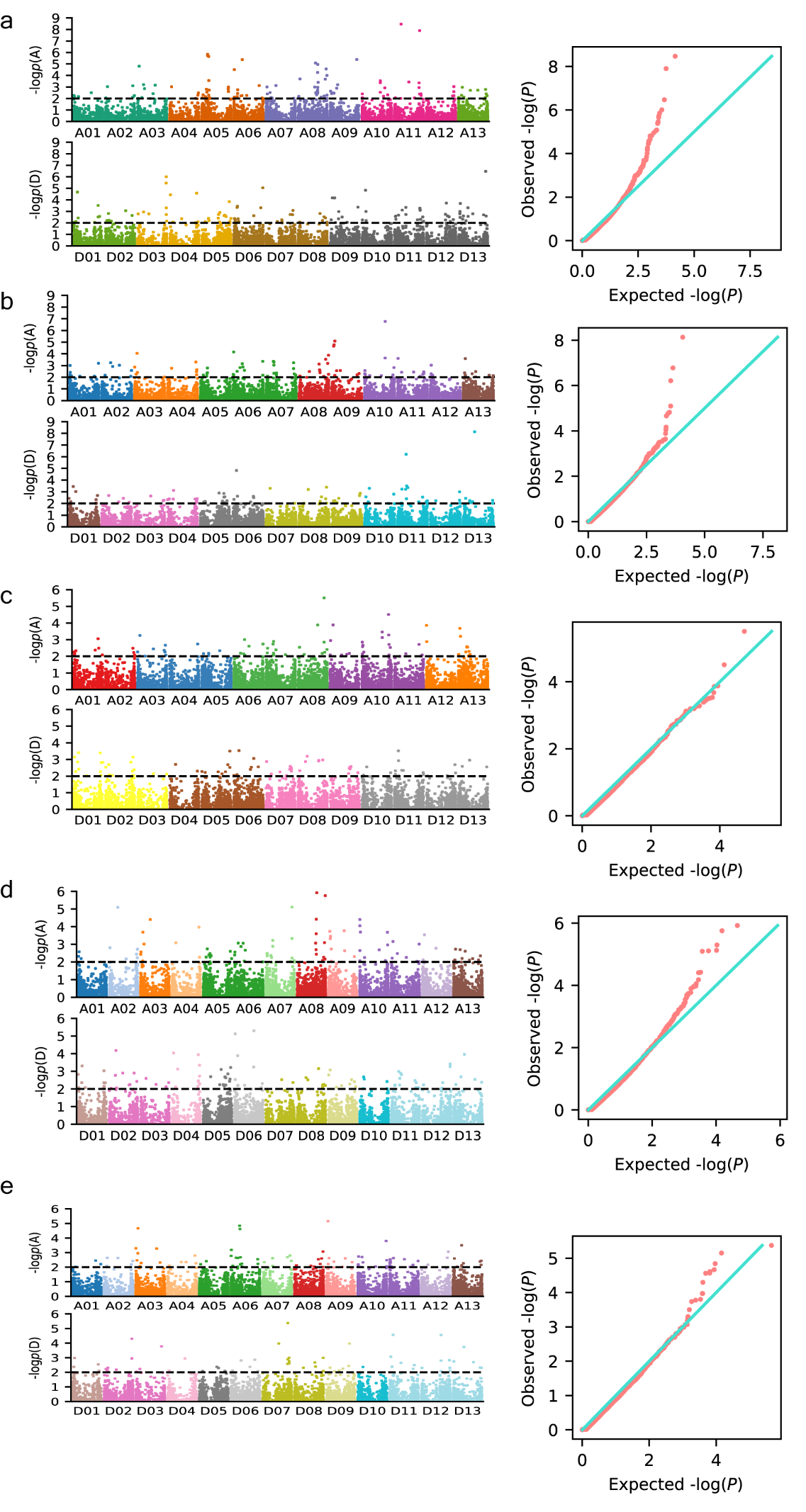


**Fig. S2** | GWAS for fiber-related traits in Ma_2018 cohort (n=419).

**a-e**. Manhattan plot and Q-Q plot for lint percentage, fiber length, fiber strength, fiber uniform and fiber elongation. Threshold of *P*-value was set as 0.01 and all selected locus were furtherly filtered as described in **Methods.**


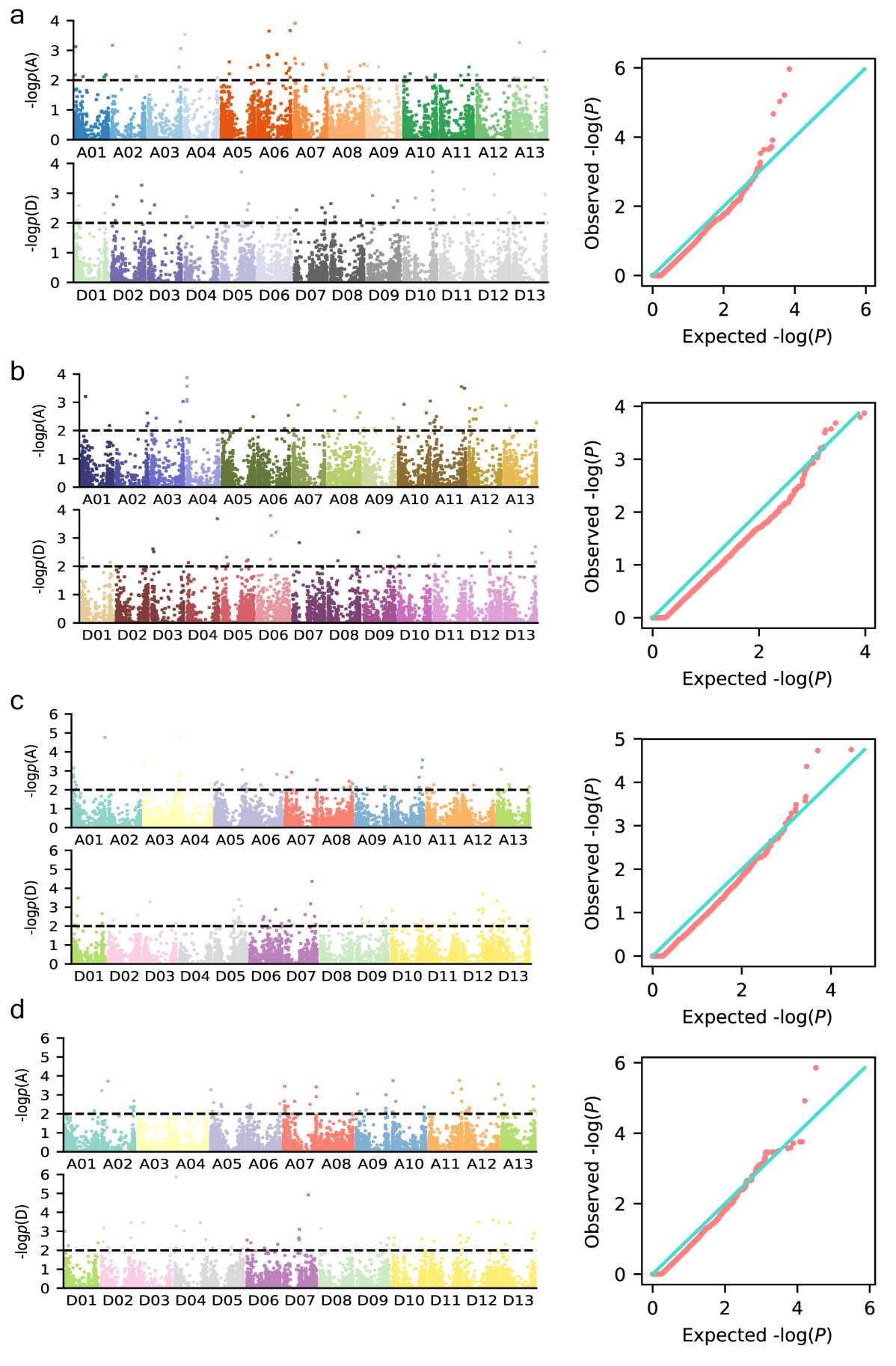


**Fig. S3** | GWAS for fiber-related traits in Wang_2017 cohort (n=169).

**a-d**. Manhattan plot and Q-Q plot for fiber length, fiber strength, fiber uniform and fiber elongation. Threshold of *P*-value was set as 0.01 and all selected locus were furtherly filtered as described in **Methods.**


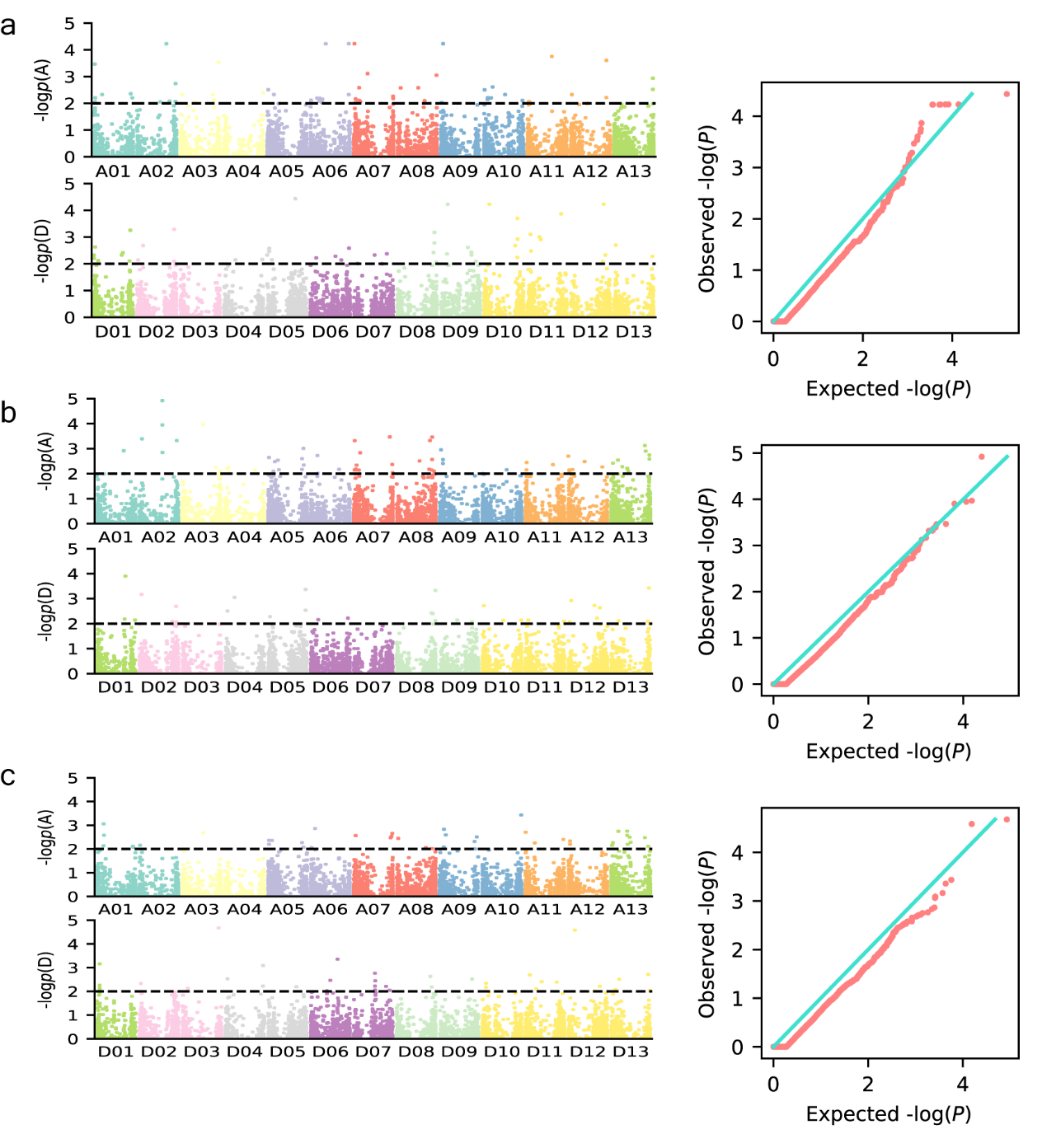


**Fig. S4** | GWAS for fiber-related traits in He_2021 cohort (n=145).

**a-c**. Manhattan plot and Q-Q plot for lint percentage, fiber length, fiber strength and fiber elongation. Threshold of *P*-value was set as 0.01 and all selected locus were furtherly filtered as described in **Methods.**


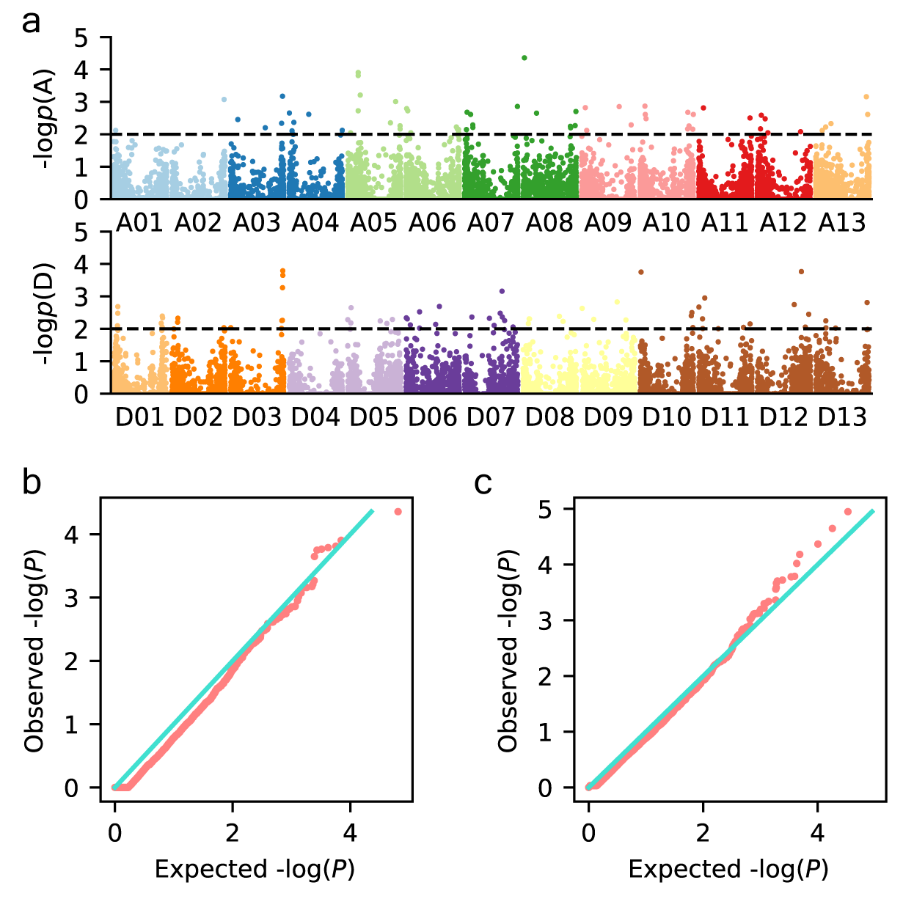


**Fig. S5** | GWAS for pathogen-resistance traits in Ma_2018 cohort (n=408) and Wang_2017 (n=208).

**a-b**. Manhattan plot and Q-Q plot for Fusarium wilt resistance in Wang_2017 cohort (n=208). **c**. Q-Q plot for GWAS on *Verticillium wilt* resistance in Ma_2018 cohort (n=408).


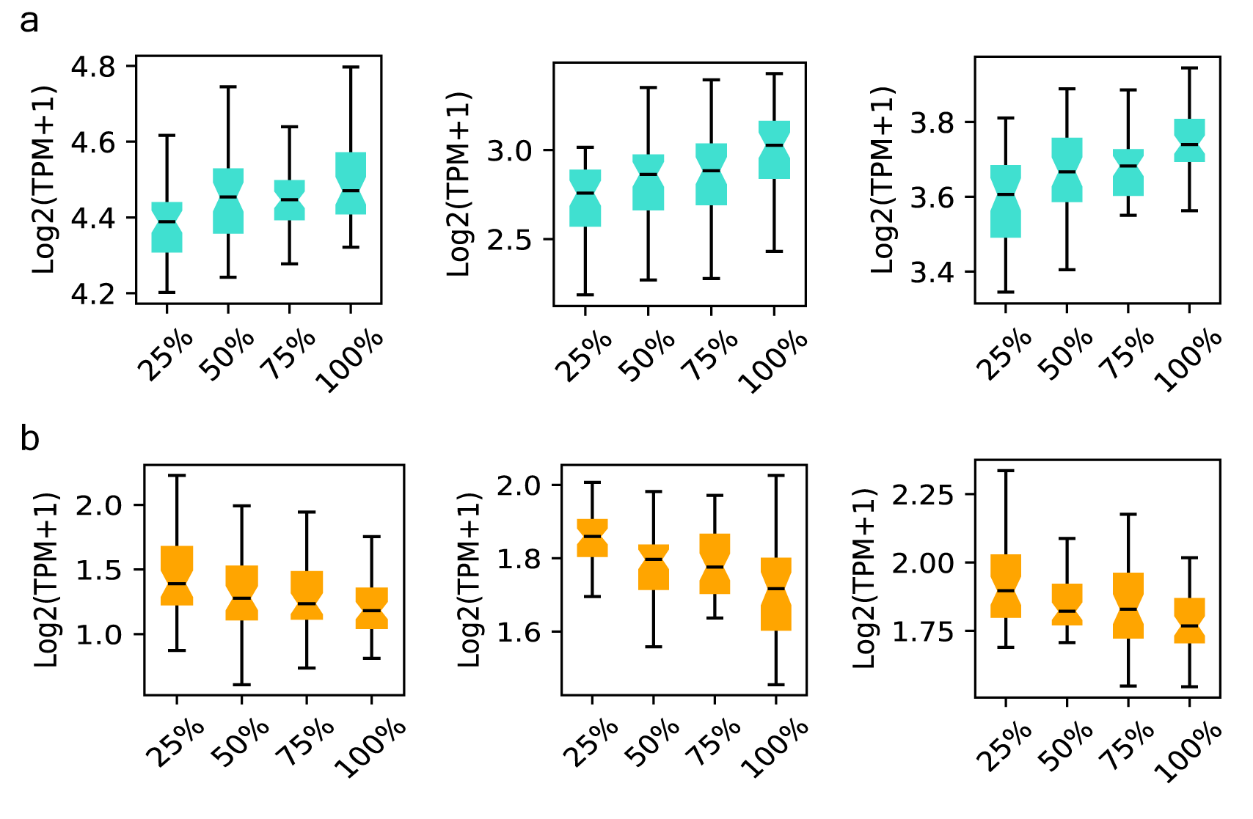


**Fig. S6** | Transcription abundance of genes from SGP triplets in Wang_2017 (n=169).

**a**. Genes in consistent SGPs related to fiber strength, fiber uniform and fiber elongation. X-axis was the rank of agronomic trait (from low to high in quartiles). Y-axis was the TPM of genes in Wang_2017 cohort. **b**. Genes in conflict SGPs related to fiber strength, fiber uniform and fiber elongation.


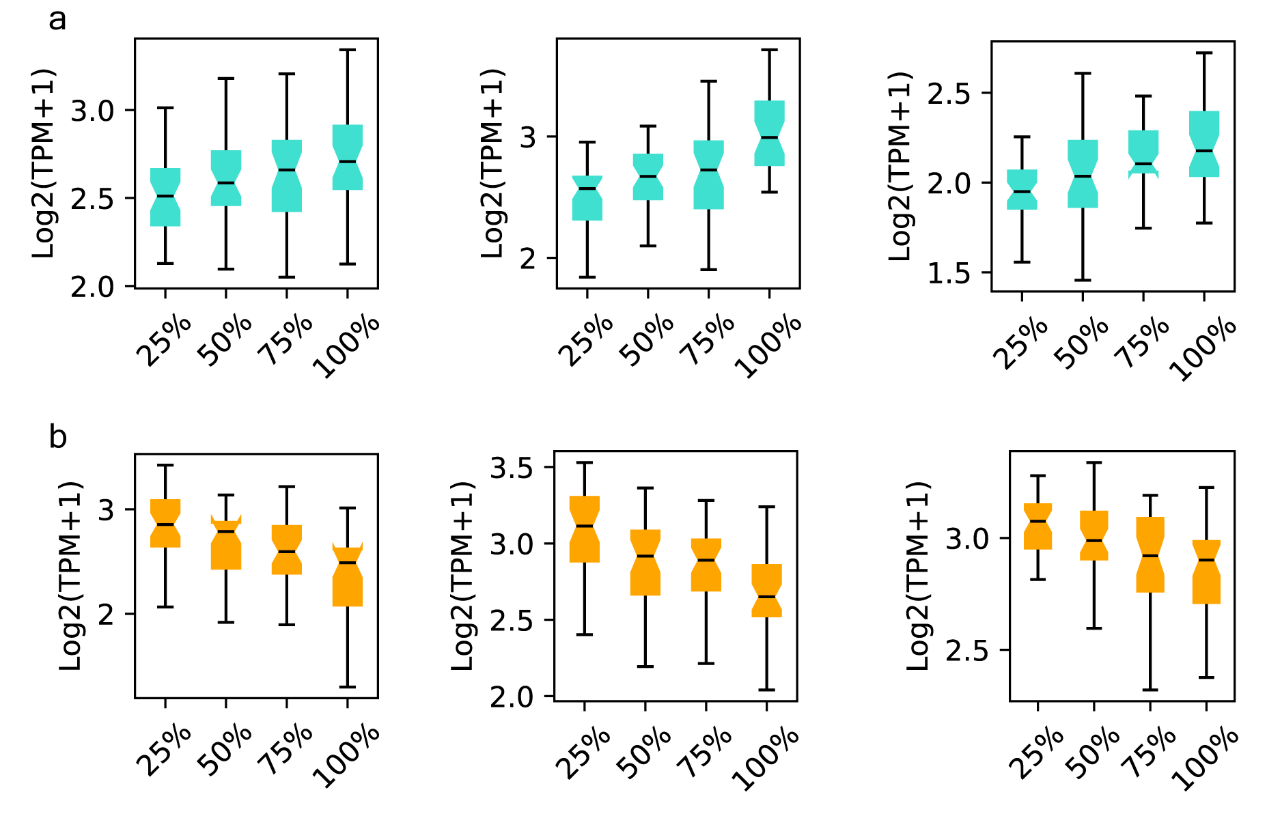


**Fig. S7** | Transcription abundance of genes from SGP triplets in He_2021 (n=145).

**a**. Genes in consistent SGPs related to fiber length, fiber strength and fiber elongation. X-axis was the rank of agronomic trait (from low to high in quartiles). Y-axis was the TPM of genes in He_2021 cohort. **b**. Genes in conflict SGPs related to fiber length, fiber strength and fiber elongation.


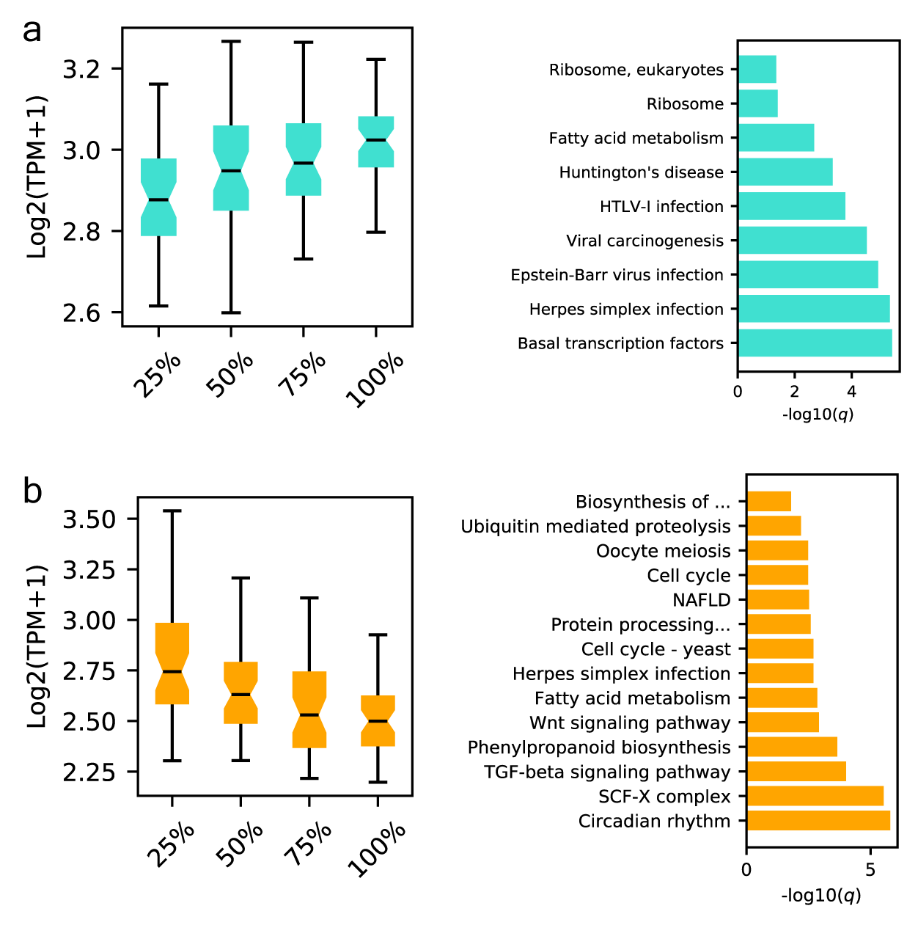


**Fig. S8** | Genes from SGP triplets about fiber length in Wang_2017 (n=169).

**a**. Left boxplot was transcription abundance of genes in consistent SGPs related to fiber length. X-axis was the rank of fiber length (from short to long in quartiles). Right barplot was the KEGG result of genes in consistent SGPs in Wang_2017 cohort. **b**. Left boxplot was Transcription abundance of genes in conflict SGPs related to fiber length. X-axis was the rank of fiber length (from short to long in quartiles). Right barplot was the KEGG result of genes in conflict SGPs in Wang_2017 cohort.


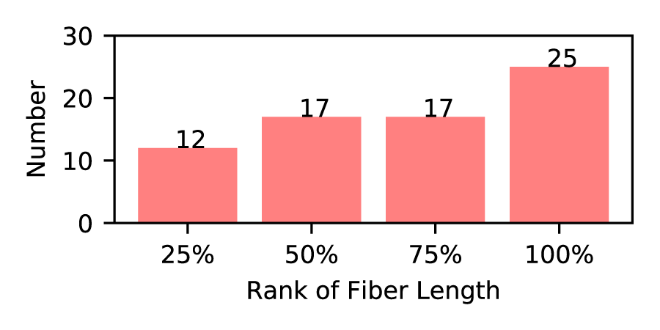


**Fig. S9** | Two elite deletions contained in Wang_2017 (n=169).

Fiber length was sorted into quartiles from short to long. The total number of 2 deletions contained by cultivars in each quartile was counted.


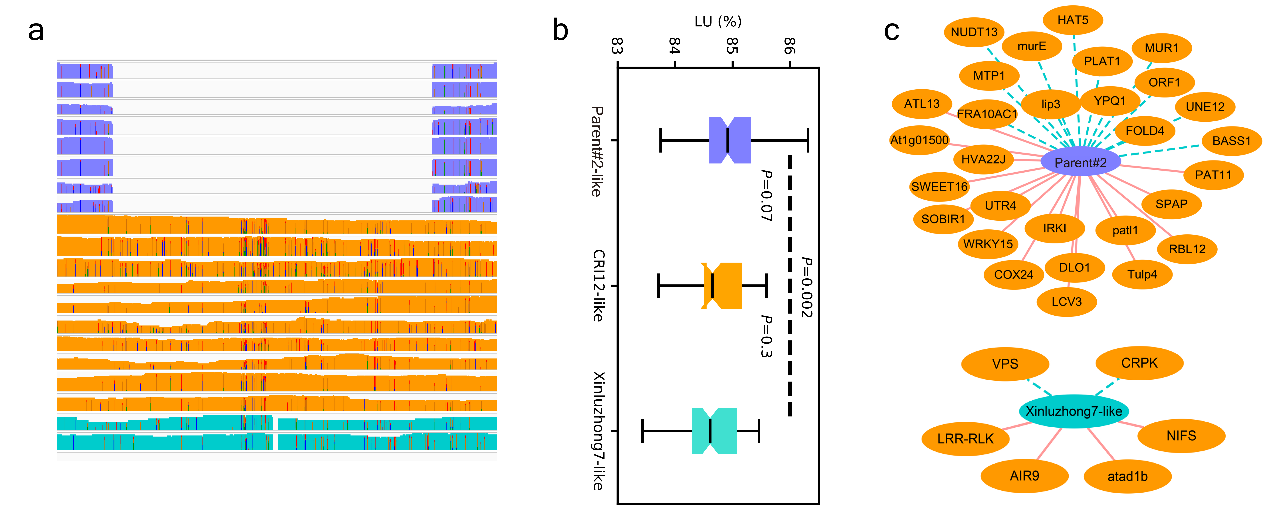


**Fig. S10** | Allelic fiber uniform-related SVs in CRI12 pedigree.

1. Allelic deletions in CRI12 pedigree. Purple lines represent for Uganda4_DEL_2661; Orange lines represent for CRI12 genotypes and blue lines represent for Xinluzhong7 genotypes. **b**. Fiber uniform of 3 genotypes. **c**. Gene regulatory network of allelic deletions.


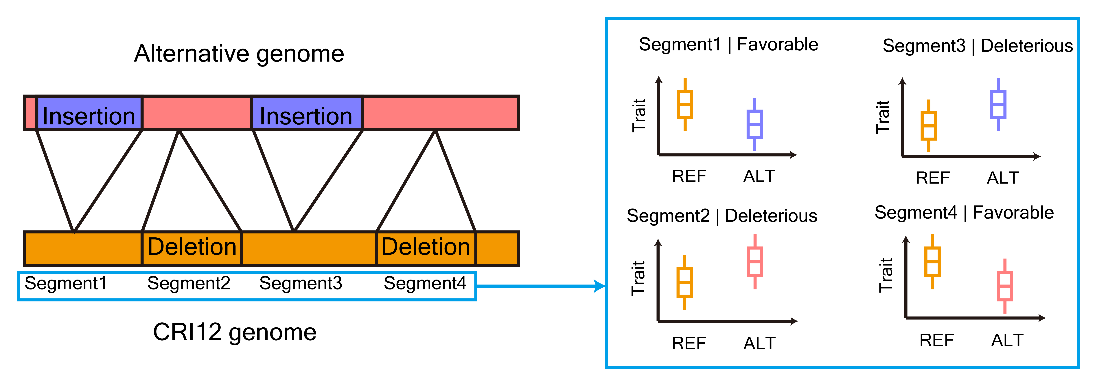


**Fig. S11** | Illustration on relationship between variations in alternative genomes and segments in CRI12 genome

Variations in alternative genomes reducing agronomic trait are mapped to corresponding favorable segments in CRI12 genome (segment1 and segment4 in CRI12 improve trait). Variations in alternative genomes improving agronomic trait are mapped to corresponding deleterious segments in CRI12 genome (segment2 and segment3 in CRI12 reduce trait).


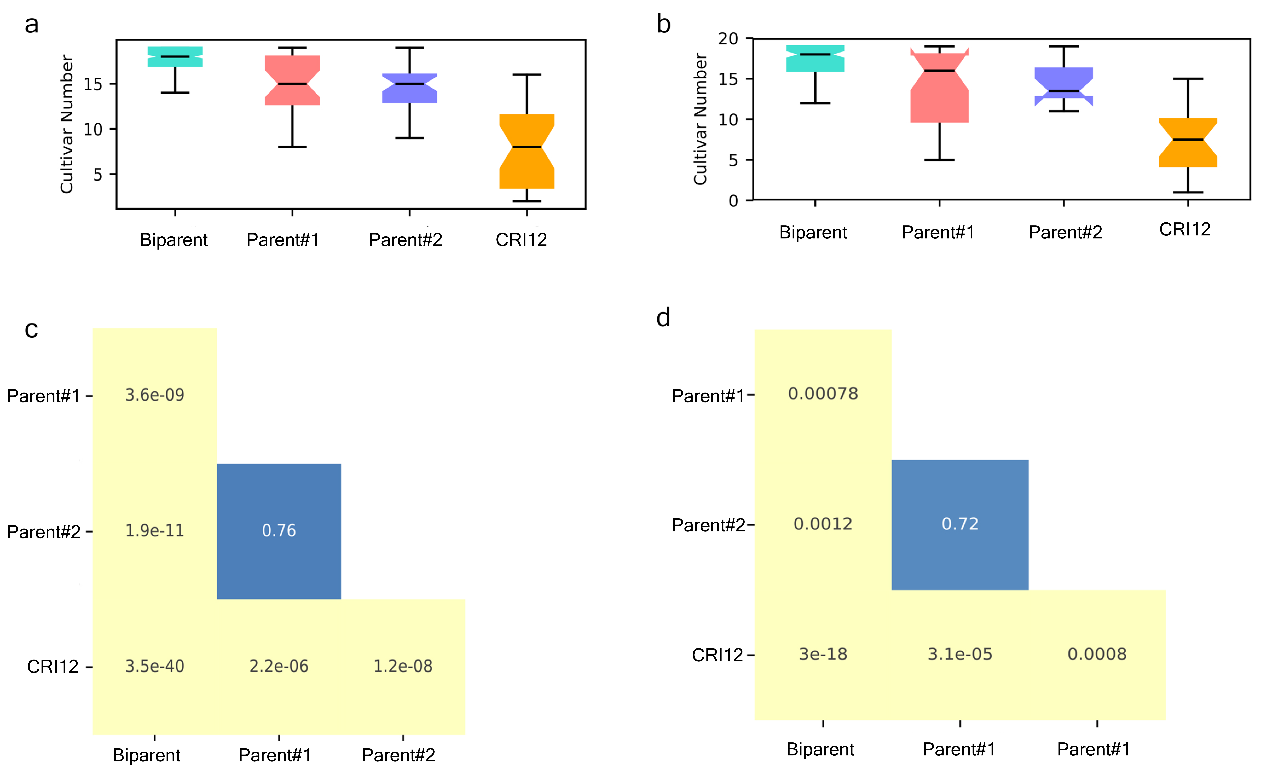


**Fig. S12** |Hereditary stability of fiber quality-related segments in CRI12 pedigree.

**a**. The boxplot of pedigree favorable segments of 4 categories. **b**. The boxplot of pedigree deleterious segments of 4 categories. **c**. t-test on hereditary stability among favorable segments of 4 categories. **d**. t-test on hereditary stability among deleterious segments of 4 categories.


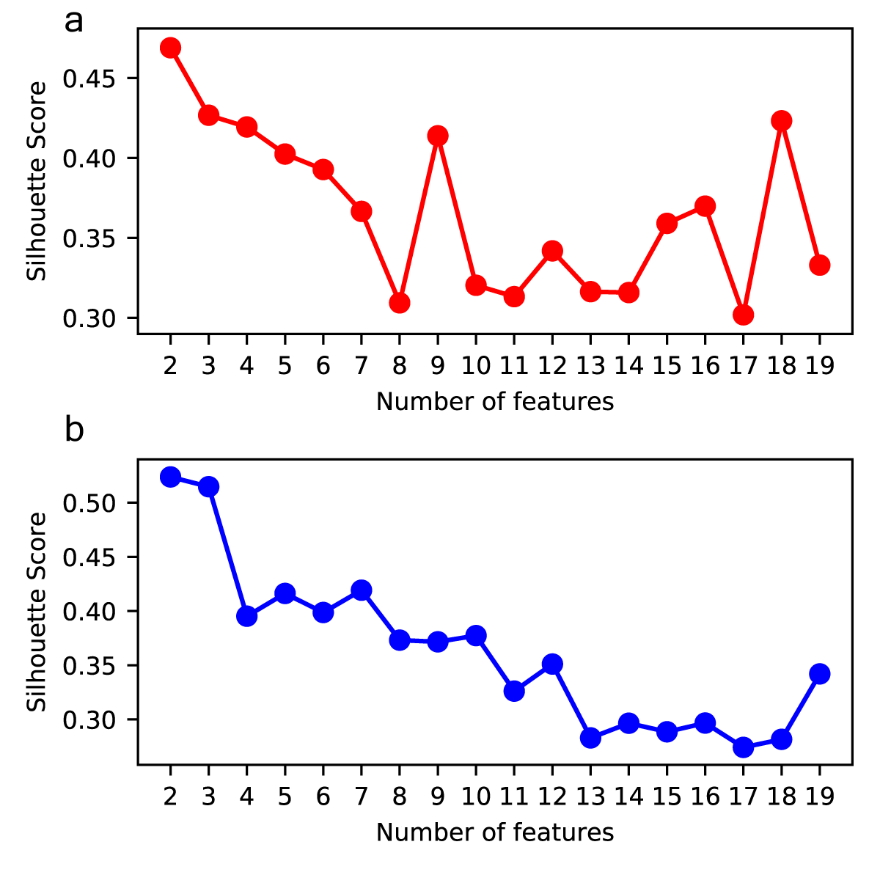


**Fig. S13** | Feature number in NMF analysis.

**a**. Selection on number of features in 70 *Fusarium wilt* resistant segments. **b**. Selection on number of features in fiber length related segments. X-axis is the number of features in NMF analysis and Y-axis is the silhouette score of sample-feature matrix.


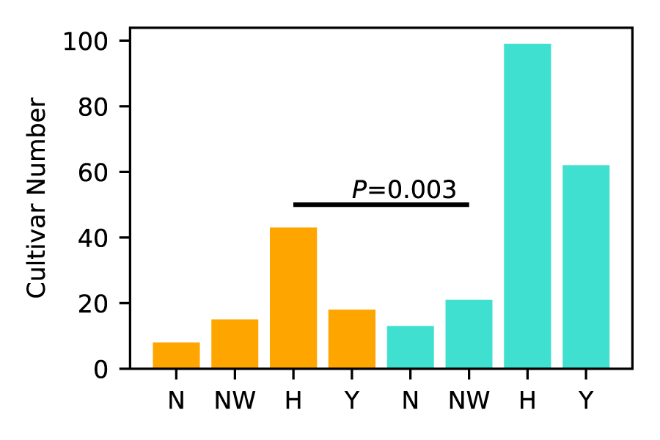


**Fig. S14** | Number of cultivars containing *GhKHCP* from 4 regions. The orange bars are numbers of cultivars with *GhKHCP* from 4 regions, while turquoise bars are numbers of cultivars without *GhKHCP* from 4 regions. Cultivars without *GhKHCP* were enriched in Yangze River region (χ^2^ test).


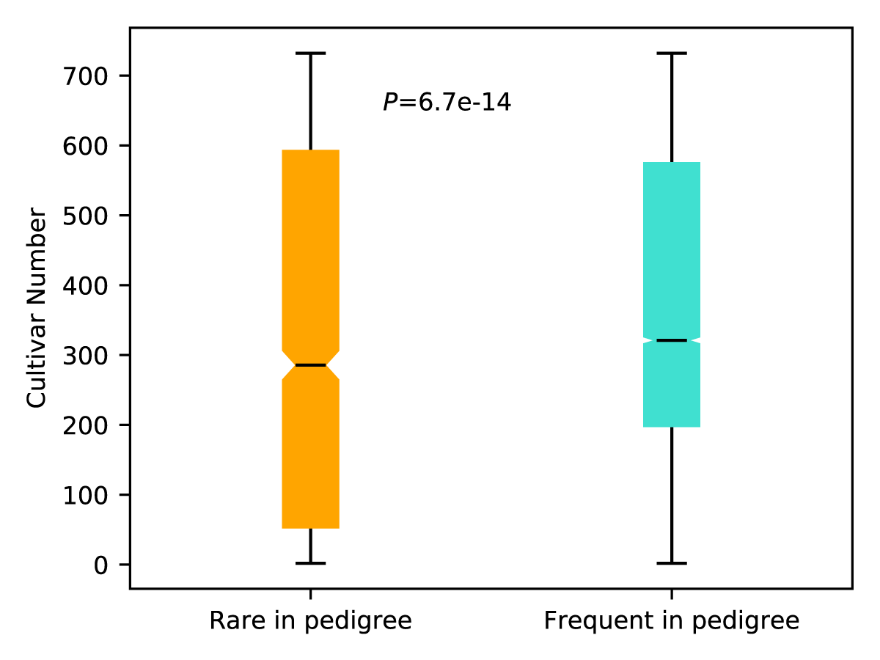


**Fig. S15** | Bottoleneck effect in CRI12 pedigree. Orange box is the present frequency of segment with low hereditary stability in 733 population. While, turquoise box is the present frequency of segment with high hereditary stability in 733 population (*P*=6.7e-14).


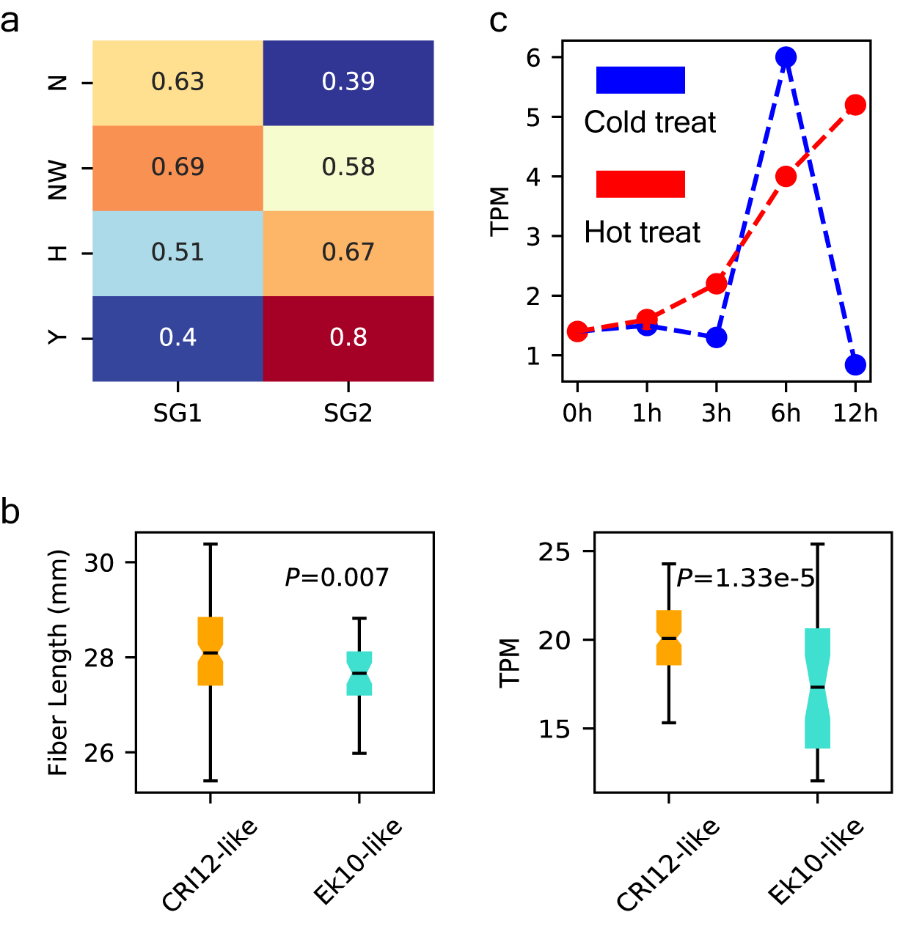


**Fig. S16** | NMF result for fiber length-related segments.

**a**. Cluster result of cultivars from 4 regions. The value in heatmap is the median feature-sample score of cultivars from each region, and 4 regions were clustered by hierarchical method. **b**. Fiber length of 2 genotypes (CRI12-like and Ekangmian10-like) and the transcription abundance of *CRI12_A09G0106* of 2 genotypes. **c**. The expression of *CRI12_A09G0106* after cold and heat treat in 1, 3, 6 and 12 hours.
