# Supplementary material for: Cotton pedigree genome reveals restriction of cultivar-driven strategy in cotton breeding
Source: Genome Biol. 2023 Dec 8;24:282. doi: 10.1186/s13059-023-03124-3 (PMC10704732; doi:10.1186/s13059-023-03124-3)
Supplement: Supplementary file 3 — Additional file 3. Review history. [file 13059_2023_3124_MOESM3_ESM.docx]

Review History

**First round of review**

**Reviewer 1**

**Were you able to assess all statistics in the manuscript, including the appropriateness of statistical tests used?**

Yes, and I have assessed the statistics in my report.

**Comments to author:**

Guoli Song and his colleagues sequenced a high-quality genome of an elite cultivar CRI12 using ONT long reads, Illumina short reads and HiC reads and also CRI12 and its parent lines's transcritomic data. CRI12 also a backbone cultivars and at least 20 cultivars have been developed based on the backbone cultivar or by a cultivar-based breeding approach. This study further de novo sequenced the CRI12 pedigree (20 members) using ONT long reads. Based on the above data/assembly genomes, they constructed a graph-based pedigree genome (GPG) of CRI12 and identified geneic variations such as PAV of the GPG. Further they compared the GPG to other three available cohorts (i.e. population genomic invesitgations), determined the genetic heretidy during the breeding programs and identified functional loci or genes by GWAS, for example resistance genes (one of them was cloned from the CRI12). In this study, they defined some new genomic items or parameters to link genomic pattern to breeding practice, such as functional segment and fingerprint segment. Based on the segment heredity, they found a low hereditary stability and geographical grouping of functional segments and very low percentage of the pedigree fingergrint segments of CRI12 in other cultivar populations (i.e. the fingerprint segments wer locked in the pedigree, not like the functional segments). Therefore they believe that the stragety of CRI12-driven breeding or backbone cultivar-dirven breeding has restriction for cotton breeding in a long-term consideration and should more focus on functional genes and from cultivar-driven shit to gene-driven breeding approach. To my knowledgement, this is the first effort to link pedigree genomic analysis results to detailed breeding strategy, i.e. challenge or argue the backbone cultivar-driven breeding approach, a very popular/typical concept of crop breeding.

Major comments:

1.The organization or presentation of this study should be improved: it is difficult ot follow, particular at the beginning or introduction part and don't know the key points they want to say. Based on my understanding, their key point should be: although the popular cultivar-driven breeding approach can generate new cultivars (just like 20 progeny cultivars were developed using CRI12 as a backbone cultivar), the approach will limit more new or elite cultivars in future because the breeding stagety will restict heredidy of good segments in whole breeding program of target crop.

Two suggestions to improve your organization: 1). In the Introduction section, introduction of CRI12 should be improved. For example, the pargraphic at 30 line of page 6 should be move to 33 line of page 5. The statement about "the cultivar-driven strategy currently applied in cotton breeding has shown limited effectiveness as the backbone cultivar will develop its own pedigree[15,16]" at line 25 should be provide more explanations. This is the key start point of this study. 2).The title of Figure 1 should be as like "Overview of this study" or Workflow of this study. The pedigree should be clearly shown, such as parent lines and progeny lines of CRI12. Figure 2a should be moved to Figure 1?

Minor comments:

1.Page should be numbered? Page 5 line 21: accessions should be lines? Line 35: should add "... of China"?

2.Results page (page 6): first paragraphy shuld be move to end of last part of Introduction section? Second paragraphy should be move to the paragraphy about introduction of CRI12?

3.In Conclusion section, "in conclusion" should be removed?

4.In figures and text, it will be better to use Parent #1 and Parent #2 to refer to two parent lines of CRI12 rather than just use Xingtai6871 and Uganda4? You can define them in your Figure 1 or Figure 2a.

**Reviewer 2**

**Were you able to assess all statistics in the manuscript, including the appropriateness of statistical tests used?**

No, I do not feel adequately qualified to assess the statistics.

**Comments to author:**

The manuscript deals with an important aspect of how we generally carry out crop improvement and points out problems found with inheritance of traits from elite lines to their progeny based on genome analyses. In addition, the authors uncovered a series of genes and DNA segments that may be useful in engineering cotton. In my opinion the methods used are adequate and the research sound. Even though I agree with many of the conclusions reached by the authors, I feel that at some points the authors seem to be cherry picking. Thus, the manuscript requires several clarifications. In addition, there are a couple of conclusions in which I disagree or find the data do not fully support them (see specific comments below). Nevertheless, the strategy is interesting and as such should call the attention of biologist in different fields, specially those interested in plant genetics and genomics. Finally, in my opinion the "discussion" is more a type of "recapitulation of the results" than an actual discussion. Although the use of language is mostly correct, I would suggest to have the manuscript corrected by a native English speaker as there are a few sentences that need to be corrected for clarity and the correct use of a few words.

Specific comments (in the order in which they appear in the manuscript):

1. Page 7; lines 7-8. Please define subgenomes.

2. Page 7; Line 35. 0.64 is not a strong Pearson Correlation. Please comment on the relevance of this value on the possibility of LTR/Gypsy transposons influencing PAVs.

3. Page 8; line 27. I suggest to modify the text from "has been shown to be" to "is".

4. Page 8; line 31. I suggest to modify "were found to be" to read "are".

5. Page 8; last line. I suggest to delete the word "the" "influenced" and "fiber length".

6. Page 9; lines 15-16. The authors mention that the expression level of GhKCR1 increased in cultivars with longer fiber. Is the increase in expression significant? Could the authors provide celar expression values? How much is the increase 10 or 20%? If so, is the change in expression sufficient to translate in biological significance? The increase shown in figure 3G does not seem to be large.

7. Page 9; line 19. I suggest to delete "observed to be".

8. Page 9; line 25. I suggest to modify the text to read "is" instead of "was proved to be".

9. Page 9; line 38. A Pearson correlation value of 0.58 is weak. Please discuss the implications of the Pearson Correlation values obtained.

10. Page 9; lines 44-50. I suggest to modify the sentence from "A pair of non-coding allelic deletions, Xinluzhong7_DEL_3213-Uganda4_DEL_2661, was focused due to their significant differential effects on fiber uniformity in the Wang_2017 cohort (P=0.002) (Additional file2: Fig. S10a and S10b)." to read "We focused on a pair of non-coding allelic deletions, Xinluzhong7_DEL_3213-Uganda4_DEL_2661, due to their significant differential effects on fiber uniformity in the Wang_2017 cohort (P=0.002) (Additional file2: Fig. S10a and S10b)."

11. Page 9; line 54. I suggest to modify the text from "The same was observed between CRI12-like and Xinluzhong7-like" to read "Similarly, CRI12-like genotypes exhibited higher fiber uniformity than Xinluzhong7-like"...

12. Page 9; lines 54-58. Do Uganda4-like genotypes exhibit significantly higher fiber uniformity than Xinluzhng7-like genotypes?

13. Page 9 lines 59-60 and Page 10 line 1. It seems risky to make such and inference based on two thresholds that were not significant.

14. Page 10; lines 15-16. Which variations were considered favorable and deleterious and why? In other words, what was considered a favorable variation and what a deleterious variation?

15. Page 10; lines 36-40. What were the basis to select segments 7, 4, 14 and 45 for further analysis?

16. Page 10 last line and page 11 first line. Why would these segments be lost in the progeny?

17. Page 11; lines 19-21. What do you mean by "Although the genetic pattern of both favorable and deleterious segments was similar to the FR-related ones"?

18. Page 12; lie 46. I suggest to add the word "which" before "encodes a KH..."

19. Page 16; line 19. I suggest to modify the text from "Among the fiber-related genes in present work" to read "Among the fiber-related genes discovered in the present work..."

20. Page 16; lines 38-42. The sentence is odd. Please clarify.

21. Legend to figure 3. Please define TPM.

**Authors Response**

**Point-by-point responses to the reviewers’ comments:**

Reviewer #1:

Guoli Song and his colleagues sequenced a high-quality genome of an elite cultivar CRI12 using ONT long reads, Illumina short reads and HiC reads and also CRI12 and its parent lines's transcritomic data. CRI12 also a backbone cultivars and at least 20 cultivars have been developed based on the backbone cultivar or by a cultivar-based breeding approach. This study further de novo sequenced the CRI12 pedigree (20 members) using ONT long reads. Based on the above data/assembly genomes, they constructed a graph-based pedigree genome (GPG) of CRI12 and identified geneic variations such as PAV of the GPG. Further they compared the GPG to other three available cohorts (i.e. population genomic invesitgations), determined the genetic heretidy during the breeding programs and identified functional loci or genes by GWAS, for example resistance genes (one of them was cloned from the CRI12). In this study, they defined some new genomic items or parameters to link genomic pattern to breeding practice, such as functional segment and fingerprint segment. Based on the segment heredity, they found a low hereditary stability and geographical grouping of functional segments and very low percentage of the pedigree fingergrint segments of CRI12 in other cultivar populations (i.e. the fingerprint segments wer locked in the pedigree, not like the functional segments). Therefore they believe that the stragety of CRI12-driven breeding or backbone cultivar-dirven breeding has restriction for cotton breeding in a long-term consideration and should more focus on functional genes and from cultivar-driven shit to gene-driven breeding approach. To my knowledgement, this is the first effort to link pedigree genomic analysis results to detailed breeding strategy, i.e. challenge or argue the backbone cultivar-driven breeding approach, a very popular/typical concept of crop breeding.

*Response to comment:*

*We appreciate your insightful and constructive comments on the manuscript. We have carefully addressed all your concerns raised in major and minor comments. We hope you satisfy with our answers. The page numbers and line numers used in response are consistent with untracked version for a clear revision, while the tracked manuscript is also provided. The blue text is the response while green text is the detailed modification text in revised manuscript.*

Major comments:

1.The organization or presentation of this study should be improved: it is difficult ot follow, particular at the beginning or introduction part and don't know the key points they want to say. Based on my understanding, their key point should be: although the popular cultivar-driven breeding approach can generate new cultivars (just like 20 progeny cultivars were developed using CRI12 as a backbone cultivar), the approach will limit more new or elite cultivars in future because the breeding stagety will restict heredidy of good segments in whole breeding program of target crop.

Two suggestions to improve your organization: 1). In the Introduction section, introduction of CRI12 should be improved. For example, the pargraphic at 30 line of page 6 should be move to 33 line of page 5. The statement about "the cultivar-driven strategy currently applied in cotton breeding has shown limited effectiveness as the backbone cultivar will develop its own pedigree[15,16]" at line 25 should be provide more explanations. This is the key start point of this study. 2).The title of Figure 1 should be as like "Overview of this study" or Workflow of this study. The pedigree should be clearly shown, such as parent lines and progeny lines of CRI12. Figure 2a should be moved to Figure 1?

*Response to major concern1:*

*We agree with your understanding and are sorry for inappropriate organization of introduction part. Your advice on organization is valuable for us. We reorganized the introduction part according to follow structure:* ***i)*** *point out the drawback of cultivar-driven strategy in first paragraph.* ***ii)*** *introduce the development of cotton genomics in second paragraph;* ***iii)*** *introduce our work. The corresponding modification was from page2 line24 to page3 line30. Introduction part is at page2 line23 to page3 line30.*

*In modified introduction, we introduce CRI12 in detail as “For instance, CRI12, as a cultivar won the first prize of the National Award for Technological Invention of China, is selected from cross lines in disease nursery and has elite integrative agronomic traits, especially for high resistance to Fusarium wilt and Verticillium wilt. This backbone cultivar is used to create many progenies, forming CRI12 pedigree by the cultivar-driven strategy[4].” (from page2 line28 to page3 line2). The limited effectiveness of cultivar-driven strategy was furtherly illustrated as “However, progenies in CRI12 pedigree do not retain elite integrative agronomic traits. Apart from CRI12, many other popular cultivars were also used to create new lines in cotton breeding history, and they exhibited limited effectiveness in cultivar-driven breeding strategy which appeared in CRI12 pedigree[5].” (page3 line2-5).*

*We have changed the title of Figure 1 as “Overview of this study” (page 27 Figure 1; Fig.R1). Figure 2a was moved to Figure 1. The name of CRI12 pedigree members and 2 parental lines were marked with “Parent#1” and “Parent#2”.*

Minor comments:

1.Page should be numbered? Page 5 line 21: accessions should be lines? Line 35: should add "... of China"?

*Response to minor comment1:*

*We are sorry for the ignorance of page numbering. In the revised manuscript, all pages and lines are numbered. We changed the word “accession” into “lines” (page2 line26) and added “of China” behind the prize name (page2 line29).*

2.Results page (page 6): first paragraphy shuld be move to end of last part of Introduction section? Second paragraphy should be move to the paragraphy about introduction of CRI12?

*Response to minor comment2:*

*Thank you for your valuable advice for the organization. We have moved the overview of the study to the last part of introduction (page3 line19-30). The detailed introduction of CRI12 has been moved to the first part of introduction (page2 line28-page3 line3).*

3.In Conclusion section, "in conclusion" should be removed?

*Response to minor comment3:*

*Thank you for your suggestion, and the word “in conclusion” has been removed (page14 line16).*

4.In figures and text, it will be better to use Parent #1 and Parent #2 to refer to two parent lines of CRI12 rather than just use Xingtai6871 and Uganda4? You can define them in your Figure 1 or Figure 2a.

*Response to minor comment4:*

*Thank you for your advice on accession display. We have defined Xingtai6871 and Uganda4 as Parent#1 and Parent#2 in Figure 1, respectively, and substituted them in all figures and text in revised manuscript.*

Reviewer #2:

The manuscript deals with an important aspect of how we generally carry out crop improvement and points out problems found with inheritance of traits from elite lines to their progeny based on genome analyses. In addition, the authors uncovered a series of genes and DNA segments that may be useful in engineering cotton. In my opinion the methods used are adequate and the research sound. Even though I agree with many of the conclusions reached by the authors, I feel that at some points the authors seem to be cherry picking. Thus, the manuscript requires several clarifications. In addition, there are a couple of conclusions in which I disagree or find the data do not fully support them (see specific comments below). Nevertheless, the strategy is interesting and as such should call the attention of biologist in different fields, specially those interested in plant genetics and genomics. Finally, in my opinion the "discussion" is more a type of "recapitulation of the results" than an actual discussion. Although the use of language is mostly correct, I would suggest to have the manuscript corrected by a native English speaker as there are a few sentences that need to be corrected for clarity and the correct use of a few words.

*Response to comments:*

*Thank you for your valuable advice and constructive comments on the manuscript. We have carefully checked and improved the English writing in the revised manuscript. We are sorry for several mistakes in manuscript which result in confusion. We have carefully addressed all your concerns raised in comments, and we hope you satisfy with our response.*

*For discussion, we rewrite it as below: i) summary of this study; ii) discussing the GPG construction strategy in this study and other previously reported genomic analysis framework; iii) discussing about the weak performance of trait prediction on fiber uniformity and fiber* *length; iv) highlinghting the innovation of this study and propose a 4G breeding strategy to capture elite genomic resource in backbone cultivar. Discussion part is at page13 to page14 line14.*

*The page numbers and line numers used in response are consistent with untracked version for a clear revision, while the tracked manuscript is also provided. The blue text is the response while green text is the detailed modification text in revised manuscript.*

*Response to specific comments:*

1. Page 7; lines 7-8. Please define subgenomes.

*Response to comment1:*

*We are sorry for the ignorance of sub-genome defination, and we have added the defination of A, D sub-genomes as “Upland cotton is an allotetraploid, and its genome (AADD) consists of sub-genome A and sub-genome D” in page 4 line19-20 in revised manuscript.*

2. Page 7; Line 35. 0.64 is not a strong Pearson Correlation. Please comment on the relevance of this value on the possibility of LTR/Gypsy transposons influencing PAVs.

*Response to comment2:*

*We agree with your opinion about the weak Pearson Correlationship. We rewrited the result as “Of these categories, LTR/Gypsy, the most abundant transposon, is highly related with PAV length in exon, promoter and interval regions (pearson correlation is 0.81, 0.72 and 0.73, respectively; 5627 of 6076 PAVs) and relatively lowly related with PAV length in intron (pearson correlation is 0.64; 449 of 6076 PAVs) (****Fig. 2e ; Additional file2: Fig. S1a****), indicating its important role in SVs proved in G. rotundifolium[22]” (page5 line4-8).*

*Because that PAVs in exon, promoter and interval regions take up over 90% of all transposon-influenced PAVs (5627 of 6076 PAVs), we think that LTR/Gypsy transposons contribute to the length of PAVs.*

3. Page 8; line 27. I suggest to modify the text from "has been shown to be" to "is".

*Response to comment3:*

*Thank you for your valuable suggestion, we have modified the text (page6 line2) in revised manuscript.*

4. Page 8; line 31. I suggest to modify "were found to be" to read "are".

*Response to comment4:*

*Thank you for your valuable suggestion, we have modified the text (page6 line4) in revised manuscript.*

5. Page 8; last line. I suggest to delete the word "the" "influenced" and "fiber length".

*Response to comment5:*

*Thank you for youradvice, we have modified the text (page6 line17-18) in revised manuscript.*

6. Page 9; lines 15-16. The authors mention that the expression level of GhKCR1 increased in cultivars with longer fiber. Is the increase in expression significant? Could the authors provide celar expression values? How much is the increase 10 or 20%? If so, is the change in expression sufficient to translate in biological significance? The increase shown in figure 3G does not seem to be large.

*Response to comment 6:*

*We are sorry for the ignorance of the t-test on expression of GhKCR1. We have performed ttest on expression of GhKCR1 and added description of significance test as “We noticed that GhKCR1A in long fiber cultivars (rank of fiber length at 50%-75% and 75%-100%) exhibited higher expression levels than short fiber cultivars (rank of fiber length at 0%-25%) with both 6.5% increase (P values were 0.024 and 0.017, respectively) (****Fig. 3g****). GhKCR1D had similar transcription patterns with GhKCR1A, and long fiber cultivars (rank of fiber length at 50%-75% and 75%-100%) exhibited 6.5% and 13% increase in expression level (P values were 0.035 and 0.0025, respectively) compared to short fiber cultivars (rank of fiber length at 0%-25%), respectively.” in revised manuscript (page6 line26-page7 line2). The divergence of GhKCR1’s expression in cultivars with longest fiber (rank of fiber length at 75%-100%) and shortest fiber (rank of fiber length at 0%-25%) is significant for both GhKCR1A and GhKCR1D (P values are 0.017 and 0.0025, respectively). The* ***Fig. R1*** *shows p values of t-test for GhKCR1s.* ***Fig.R2*** *(same as* ***Fig. 3g****) shows clear expression of GhKCR1.*

**a**	**b**

Fig. R1 | P values of t-test on expression of *GhKCR1*. a. t-test for *GhKCR1A*. X-ticks show cultivars at 3 fiber length levels: 0-25%, 25%-50% and 25%-75%. b. t-test for *GhKCR1D*.

Fig. R2 | Expression of *GhKCR1*. Top boxplot shows expression of *GhKCR1A* and bottom boxplot shows expression of *GhKCR1D*. The median value is marked beyond each box.

*For expression of GhKCR1A, although only cultivars at 75-100% and 50%-75% rank of fiber length show significant increase compared with those at 0%-25%, the gradual increase of median expression level is still observed among 4 cultivar groups. Morever, a gradual increase of p values in t-test between cultivars at 75%-100% rank of fiber length and those at other levels was observed (0.017, 0.5 and 0.93 for 0%-25%|75%-100%, 25%-50%|75%-100% and 50%-75%|75%-100%, respectively), indicating divergence of GhKCR1A expression is gradually degraded with decrease of fiber length divergence (****Fig. R1a****). Similar expression and p value patterns were also oberved in GhKCR1D (****Fig. R1b****).*

7. Page 9; line 19. I suggest to delete "observed to be".

*Response to comment7:*

*We thank you for your suggestion and, it was corrected in revised manuscript (page7 line4).*

8. Page 9; line 25. I suggest to modify the text to read "is" instead of "was proved to be".

*Response to comment8:*

*We thank you for your suggestion and, it was corrected in revised manuscript (page7 line7).*

9. Page 9; line 38. A Pearson correlation value of 0.58 is weak. Please discuss the implications of the Pearson Correlation values obtained.

*Response to comment9:*

*Your concern about weak Pearson Correlation is precise. We rewrite the result about model performance as “The Pearson correlation coefficient between predicted values and true values of multiple traits ranged from 0.58 to 0.85 (P values range from 1.09e-52 to 1.38e-129). These results show that except for fiber uniformity and fiber length, the other agronomic traits could be predicted reliably by models constructed based on functional PAVs. (****Fig. 3i****).” in revised manuscript (page7 line12-15). We also discuss this result in discussion part as “It is worth noting that for these two traits, the predicted values showed a weak pearson correlation with the actual traits. Because GPG only considers variations in pedigree, it may overlook variations related to fiber uniformity and fiber length compared to a cotton pan-genome including 182,593 SVs[18]” (page13 line25-28).*

10. Page 9; lines 44-50. I suggest to modify the sentence from "A pair of non-coding allelic deletions, Xinluzhong7_DEL_3213-Uganda4_DEL_2661, was focused due to their significant differential effects on fiber uniformity in the Wang_2017 cohort (P=0.002) (Additional file2: Fig. S10a and S10b)." to read "We focused on a pair of non-coding allelic deletions, Xinluzhong7_DEL_3213-Uganda4_DEL_2661, due to their significant differential effects on fiber uniformity in the Wang_2017 cohort (P=0.002) (Additional file2: Fig. S10a and S10b)."

*Response to comment10:*

*We thank you for your suggestion and the corresponding sentence has been modified at page7 line17-20.*

11. Page 9; line 54. I suggest to modify the text from "The same was observed between CRI12-like and Xinluzhong7-like" to read "Similarly, CRI12-like genotypes exhibited higher fiber uniformity than Xinluzhong7-like"...

*Response to comment 11:*

*We thank you for your suggestion. Because we have reorganized this part, the corresponding part in revised manuscript is “However, compared to CRI12-like genotypes, both Parent#2-like and Xinluzhong7-like genotypes did not reach the significant divergence threshold in fiber uniformity (P=0.07 and P=0.3 for Parent#2-like and Xinluzhong7-like genotypes, respectively)” (page7 line20-23). Parent#2 represents Uganda4.*

12. Page 9; lines 54-58. Do Uganda4-like genotypes exhibit significantly higher fiber uniformity than Xinluzhng7-like genotypes?

*Response to comment 12:*

*We are sorry for ignorance of marked p value in t-test between 2 genotypes in text. In revised manuscript, we have added the description of t-test between 2 genotypes as “On this locus, Parent#2-like genotypes had significantly higher fiber uniformity than Xinluzhng7-like genotypes (P=0.002) (****Additional file2: Fig. S10a*** *and* ***S10b****).” (page7 line19-20).*

13. Page 9 lines 59-60 and Page 10 line 1. It seems risky to make such and inference based on two thresholds that were not significant.

*Response to comment13:*

*Your concern about the inference is precise. We delete the risky inference and rewrite the comments on the fiber uniformity comparsion between 2 genotypes as “However, compared to CRI12-like genotypes, both Parent#2-like and Xinluzhong7-like genotypes did not reach the significant divergence threshold in fiber uniformity (P=0.07 and P=0.3 for Parent#2-like and Xinluzhong7-like genotypes, respectively), resulting in their dismissal according to the threshold of PAV-GWAS described in the Methods (****Additional file2: Fig. S10a*** *and* ***S10b****).” in revised manuscript (page7 line20-24).*

14. Page 10; lines 15-16. Which variations were considered favorable and deleterious and why? In other words, what was considered a favorable variation and what a deleterious variation?

*Response to comment14:*

*We are sorry for vague illustration for favorable|deleterious segments in text. In revised manuscript, we added the explanation about favorable|deleterious segments as “Taking the phenotype effects into consideration, we defined a deleterious|favorable segment in CRI12 genome if the variation in alternative genome happend on corresponding locus could improve|reduce agronomic traits (****Additional file2: Fig. S11****).” (page8 line3-5). The graphical illustration is available in Fig. R3 (same as Fig. S11).*

Fig. R3 | Illustration for functional segments in CRI12 genome. The pedigree members (Alternative genome) were aligned to CRI12 genome (Reference genome) to generate variations (insertions and deletions). If cultivars with the variation have degraded agronomic trait, that means the corresponding segment in CRI12 genome (reference genome) could improve agronomic trait and is defined as favorable segments in CRI12 genome (Segment1 and Segment4 in Fig. R3).

15. Page 10; lines 36-40. What were the basis to select segments 7, 4, 14 and 45 for further analysis?

*Response to comment15:*

*We are sorry for vague description about segments selection. Through PAV-GWAS, we have identified 70 FR-related variations and their corresponding segments in CRI12 genome were selected for further analysis. In revised manuscript, we describe this sentence as “Among these 70 segments, 4, 7, 14, and 45 of them were assigned as Parent#1-inherited, Parent#2-inherited, CRI12-specific, and Biparental segments, respectively (Additional file1: Table S20).” in revised manuscript (page8 line16-18).*

16. Page 10 last line and page 11 first line. Why would these segments be lost in the progeny?

*Response to comment16:*

*In this study, we observed the low hereditary stability of functional segments in backbone cultivar, we infered that low hereditary stability results from inadequate genetic selection as described in page9 line13-16, “In the genetic trajectory of biparent segments in pedigree formation, we inferred that at least three rounds of selection for hereditary stability had occurred (from progenitor cultivars to Parent#1|Parent#2, from Parent#1|Parent#2 to CRI12, and from CRI12 to progenies). We concluded that only segments of high hereditary stability could be retained in progenies.”*

17. Page 11; lines 19-21. What do you mean by "Although the genetic pattern of both favorable and deleterious segments was similar to the FR-related ones"?

*Response to comment17:*

*We are sorry for the vague description. Here, we found that genetic pattern of CRI12-specific segments influencing fiber quality (both favorable and deleterious segments) is similar to CRI12-specific segments in FR-related segments. We rewrite this sentence as “Similar to genetic pattern of 70 FR-related segments, both favorable and deleterious CRI12-specific segments presented low hereditary stability (P value ranged from 0.0008 to 3.5e-40) (Fig. 4e; Additional file2: Fig. S12).”*

18. Page 12; lie 46. I suggest to add the word "which" before "encodes a KH..."

*Response to comment18:*

*Thank you for your suggestion, we have corrected it at page10 line21.*

19. Page 16; line 19. I suggest to modify the text from "Among the fiber-related genes in present work" to read "Among the fiber-related genes discovered in the present work..."

*Response to comment19:*

*Thank you for your valuable advice, however, in the new discussion part, we have deleted discussion about fiber-related genes.*

20. Page 16; lines 38-42. The sentence is odd. Please clarify.

*Response to comment20:*

*Thank you for your suggestion. In new discussion, this sentence has been deleted.*

21. Legend to figure 3. Please define TPM.

*Response to comment21:*

*Thank you for your suggestion. We have added the defination of TPM in figure legend of Fig. 3 as “Y-axes are TPM (Transcripts per million mapped reads) of GhKCR1A and GhKCR1D, respectively.”*

**Second round of review**

**Reviewer 1**

They have significantly improved their manuscript accordingly and no further comments.

I have carefully read the revised version by Song and his colleagues and their respones to our comments. Firstly, I am happy with their responses to my concerns/comments about their manuscript. I also read reviewer2’s comments carefully and their responses. Generally, the reviewer2 provided positive comments for the manuscript, such as “the authors uncovered a series of genes and DNA segments that may be useful in engineering cotton” and “the methods used are adequate and the research sound”. And he/she also agree with many of the conclusions reached by the authors. In my opinion, the points or conclusions argued by reviewer2 have been well anwsered or fixed by the authers in the revised version.

> The manuscript’s topic involves an important issue of breeding program and they provided a new view on breeding stratege of crops, which, in my opionion, is valuable for breeders.
